# Generating tertiary protein structures via interpretable graph variational autoencoders

**DOI:** 10.1093/bioadv/vbab036

**Published:** 2021-11-29

**Authors:** Xiaojie Guo, Yuanqi Du, Sivani Tadepalli, Liang Zhao, Amarda Shehu

**Affiliations:** 1 Department of Information Sciences and Technology, George Mason University, Fairfax, VA 22030, USA; 2 Department of Computer Science, George Mason University, Fairfax, VA 22030, USA; 3 Department of Computer Science, Emory University, Atlanta, GA 30322, USA; 4 Department of Bioengineering, George Mason University, Fairfax, VA 22030, USA

## Abstract

**Motivation:**

Modeling the structural plasticity of protein molecules remains challenging. Most research has focused on obtaining one biologically active structure. This includes the recent AlphaFold2 that has been hailed as a breakthrough for protein modeling. Computing one structure does not suffice to understand how proteins modulate their interactions and even evade our immune system. Revealing the structure space available to a protein remains challenging. Data-driven approaches that learn to generate tertiary structures are increasingly garnering attention. These approaches exploit the ability to represent tertiary structures as contact or distance maps and make direct analogies with images to harness convolution-based generative adversarial frameworks from computer vision. Since such opportunistic analogies do not allow capturing highly structured data, current deep models struggle to generate physically realistic tertiary structures.

**Results:**

We present novel deep generative models that build upon the graph variational autoencoder framework. In contrast to existing literature, we represent tertiary structures as ‘contact’ graphs, which allow us to leverage graph-generative deep learning. Our models are able to capture rich, local and distal constraints and additionally compute disentangled latent representations that reveal the impact of individual latent factors. This elucidates what the factors control and makes our models more interpretable. Rigorous comparative evaluation along various metrics shows that the models, we propose advance the state-of-the-art. While there is still much ground to cover, the work presented here is an important first step, and graph-generative frameworks promise to get us to our goal of unraveling the exquisite structural complexity of protein molecules.

**Availability and implementation:**

Code is available at https://github.com/anonymous1025/CO-VAE.

**Supplementary information:**

Supplementary data are available at *Bioinformatics Advances* online.

## 1 Introduction

Decades of research have demonstrated that protein molecules are inherently dynamic and leverage their structural plasticity to interact with multiple partners in the cell (Boehr and Wright, 2008; Boehr *et al.*, 2009) and even evade our immune system. A growing number of studies have shown structural plasticity in action: the receptor-binding domain of the severe acute respiratory syndrome coronavirus 2 (SARS-CoV-2) spike glycoprotein, assumes a closed structure in its stealth mode, where it hides from our antibodies, and a partially open structure in its offense mode, where it binds to the human Angiotensin-converting enzyme 2 receptor, thus mediating viral entry in human host cells (Henderson *et al.*, 2020; Majumder *et al.*, 2021; Tian and Tao, 2021).

Capturing protein structural plasticity remains challenging for both wet and dry laboratories (Nussinov *et al.*, 2019), primarily due to the disparate temporal scales at which conversions between tertiary structures happen and the presence of short-lived structural states. Computational approaches harness specific knowledge about a protein in the form of reaction coordinates and/or existing structure data, reduce the representation detail of a tertiary structure, utilize specialized energy functions, and/or leverage specialized techniques to expedite simulations based on Newtonian mechanics or enhance the sampling of complementary, optimization-based algorithms (Maximova *et al.*, 2016). Currently, computational approaches do not generalize well. In contrast, methods that focus on computing one structure have shown better generalizability. For decades, improvements came from a combination of better fragment libraries, more accurate structure scoring functions, and more powerful optimization algorithms seeking local minima of scoring functions. Recent methods based on deep learning have shown rapid improvements. The performance of AlphaFold2 (Jumper *et al.*, 2021) in CASP14 suggests that an important milestone has been achieved in single-structure prediction.

The question of whether a computational approach can reveal the diversity of structures employed by a protein molecule to regulate molecular interactions still stands. Methods like AlphaFold2 and others do not provide a broad view of the structure space. On the other hand, balancing between exploration and exploitation in a vast, high-dimensional structure space (with hundreds or thousands of dimensions even for a protein with a hundred or fewer amino acids) is a fundamental algorithmic challenge. Prompted by the re-emergence of neural networks in data-rich domains, including molecular structure biology, researchers are now asking an interesting question: can we learn how to generate structures of protein molecules from existing structural data?

A recent survey (Hoseini *et al.*, 2021) overviews the current landscape of generative models for protein structure generation and reveals that, while preliminary results seem promising, much confusion abounds. Many authors confound their contributions to the body of work in protein modeling and arbitrarily frame their work as either falling under protein design, protein folding or protein structure prediction. The problem of protein design asks the question of what amino acid sequences would populate a given tertiary structure most optimally (under some definition of optimality that is related to sequence-structure scoring functions)? The problem of protein folding asks about the physical process that shows how an unstructured chain of amino acids finds its way to one or more stable and/or semi-stable structures. For instance, AlphaFold2 does not address protein folding. Its contribution is in protein structure prediction and in the narrow setting of one sequence, one structure. Generative models that claim to address protein structure prediction, even if interpreted more broadly to contribute to the larger problem of revealing the protein structure space, do not consider the specific amino acid sequence. In fact, the majority of deep generative models ask a rather modest question: given tertiary structures (with some choice of representation), can the model generate protein-like (physically realistic) tertiary structures without any particular amino acid sequence in mind?

Though modest, the above question is an important milestone to demonstrate convincingly. Work in Hoseini *et al.* (2021) reveals that there is much ground to cover, even when clarifying the question. Current deep generative methods predominantly exploit the ability to represent tertiary structures as contact maps or distance matrices, which encode the spatial proximity of pairs of amino acids (often using alpha carbon or backbone atoms to represent amino acids). Some earlier works employed dihedral angles to represent protein structures; however, as such representations are under-constrained, the generated structures contained steric clashes and failed short in other characteristics attributed to physically realistic structures. The reason that contact maps or distance matrices have become popular is that they capture more constraints that are inherent in a tertiary structure. They also allow researchers to make direct analogies with (pixel maps) images and so leverage convolution-based generative adversarial frameworks from computer vision. A detailed review of these methods is beyond the scope of this article. However, as the recent review in Hoseini *et al.* (2021) relates, existing methods build over the generative adversarial network (GAN) framework, in which a discriminator network helps a generator network to learn the implicit distribution of contact maps (or distance matrices) in the training dataset.

Across the landscape of deep generative methods, the quality of the generated contact or distance maps is varied. In fact, most studies do not take a deep dive into evaluating key aspects of the quality of the generated maps. Work in Rahman *et al.* (2021) exposes issues with existing, state-of-the-art (SOTA) methods in capturing all the intrinsic structural constraints in a tertiary structure. Very recent SOTA methods leverage the loss function as the mechanism by which to focus the network on what to learn, such as the symmetry of contact maps (Yang *et al.*, 2020), the few long-range distances (in the 4–16 Å range) in distance maps over the abundant short-range or blank cells (Ding and Gong, 2020), and more (Hoseini *et al.*, 2021). Work in Rahman *et al.* (2021) shows that training GANs is fraught with instability and impacts the quality of generated data.

Noticeably, methods building over the variational autoencoder (VAE) framework have been absent. In this article, we posit that VAEs present an interesting complementary framework and warrant further attention. In particular, we posit that they allow bringing over recent developments in deep learning that better capture the rich structure (and constraints) in tertiary structures (and, more broadly, molecular structures). In particular, we maintain that a central reason behind the current difficulties of GAN-based methods to compute physically realistic tertiary structures is that they leverage image convolution. However, tertiary structures are three-dimensional objects, and proximity among the building blocks extends from sequence to three-dimensional space. Graph-based representations allow capturing spatial proximity, and bringing them over to structure generation is one of the main contributions in this article. Leveraging developments in graph-generative deep learning, this work represents a tertiary structure as a *contact graph* and learns over the space of known contact graphs (corresponding to different tertiary structures) to generate more contact graphs. In particular, we build over a graph-generative VAE framework and propose several graph-generative VAE models. We posit that VAE-based models are more powerful; by exposing latent factors, they permit more understanding of what the models have learned. Specifically, building on our work on disentangled representations in other domains, we propose here disentangled graph-generative VAE models, which reveal the impact of individual latent factors. This aids our understanding of what the factors control and so makes our models more interpretable.

## 2 Methods

**Fig. 1.**
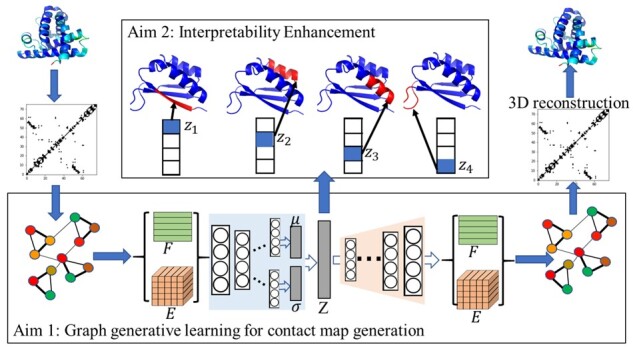
Overall schematic of the proposed generative learning framework. A contact map is extracted from a given tertiary structure and is then converted into a contact graph. Contact graphs in the training dataset are distilled to populate node and edge attribute data, which are then passed to an encoder network to learn a disentangled representation. New node and edge attribute data are then obtained from the decoder network. These are used to obtain a new contact graph, which is then converted into a contact map, which is used in turn to obtain a new tertiary structure

Figure 1 summarizes the overall approach. The schematic shows that tertiary structures in the training dataset are first represented as contact maps, which are then converted to contact graphs. This dataset is used to train a graph VAE, summarized in the bottom panel. The output of the trained model is generated contact graphs, from which one can then recover tertiary structures in a pipeline, using established methods that recover tertiary structures from contact maps (as we show here). The middle top panel of Figure 1 shows conceptually that the learned latent factors capture various aspects of a tertiary structure, and by varying them one obtains insight into what the model has learned. In the interest of space, the exposition of our approach balances between allowing the reader to understand the approach in its entirety while relating the novel methodological components. We assume some familiarity with VAE architecture and graph VAEs (Kingma and Welling, 2014).

### 2.1 Problem formulation and setup

The tertiary structures in the training dataset have atomistic detail; i.e. Cartesian coordinates are available for every atom. In keeping with convention, we extract the contact map from a given tertiary structure by making use only of the Cartesian coordinates of the central CA atoms (there is one in each amino acid). Each CA-only structure is converted into a contact graph. In summary, if the Euclidean distance between the CA atoms of amino acid *i* and *j* (with amino acids numbered 1 through *n* from the N- to the C-terminus) is no higher than 8 Å, an edge is placed between vertices representing amino acids *i* and *j*. In this way, a dataset of tertiary structures is converted into a dataset of contact graphs. We note that the threshold of 8 Åis predominantly used in contact map research literature, based on early work and analysis by Vendruscolo *et al.* (1997).

We employ the concept of a contact map graph, which can be computed as described from a given tertiary structure. Inversely, a tertiary structure can be recovered from a contact map. We do so by using CONFOLD (Adhikari *et al.*, 2015). Hence, the contact map is a graph-based representation of a tertiary structure that effectively embeds the three-dimensional Cartesian space into a two-dimensional space. Specifically, in our approach, a contact map is denoted as a graph with *N* nodes, where each node is an amino acid, and an edge between a pair of nodes encodes the adjacency relation between them.

We denote such an undirected graph as G=(E,F), which is associated with its edge attribute tensor $E\in {\mathbb{R}}^{N\times N\times L_{1}}$, where *L*_1_ denotes the number of edge attributes. In our implementations in this article, $L_{1}=1$, and so the edge attribute tensor $E\in {\mathbb{R}}^{N\times N}$, where $E_{i,j}=1$ records an interaction (contact) between vertices *i* and *j*, and $E_{i,j}=0$ otherwise. We also consider each node to have a connection to itself. The node attribute matrix is $F\in {\mathbb{R}}^{N\times L_{2}}$ and denotes the attributes of each amino acid by hot vector embedding, where *L*_2_ is the number of attributes. Node attributes can store not just the identities of the amino acids, but also their Position-Specific Scoring Matrix profile, their solvent accessibility and secondary structure as derived from a given tertiary structure. The edge attributes can encode additional information about contacts, such as their exact distance and/or contact predicted for that pair of amino acids (nodes) from sequence information alone. In our experiments, $L_{2}=20$, which corresponds to the number of all the classic amino acid types, and $L_{1}=1$, which corresponds to the existence of the contacts.

### 2.2 Deep protein contact graph VAE

In order to evaluate the fundamental premise that graph VAEs are a promising framework for (protein) contact graph generation, we design what we refer to as the CO-VAE model, which stands for Deep (Protein) Contact Graph Variational Autoencoder (CO-VAE). This model serves as a baseline, as it does not contain the disentanglement mechanism, we propose in a more sophisticated model.

Let us first summarize CO-VAE. The overall CO-VAE architecture consists of a graph encoder and a graph decoder, which are trained by optimizing the variational lower bound loss with respect to the variational parameters:

(1) $$\ell =E_{q(Z|F,E)}[\text{log}\;p(E,F|Z)]-KL[q(Z|F,E)||p(Z)].$$

In the above equation, $Z\in {\mathbb{R}}^{1\times H}$ are the stochastic latent variables vectors. *E* and *F* are the edge and node attribute tensors, respectively. The first item on the right-hand-side is the expected reconstruction loss of the generated contact graphs. The second term is a regularization term that encourages the approximate posterior q(Z|F,E) to be close to the prior *p*(*Z*). Specifically, the term measures the KL[q(·)||p(·)] Kullback–Leibler divergence (Kullback, 1997) between q(·) and p(·). This term enforces that the inferred latent vector is close to the prior distribution. $p(Z)=\prod_{i}p(z_{i})=\prod_{i}N(z_{i}|0,1)$ is a Gaussian prior; we note that in a VAE, the prior over the latent variables is modeled as a centered isotropic multivariate Gaussian.

For the encoder part q(Z|F,E), we take a simple inference model parameterized by a two-layer Graph Convolution Neural Network (GCN) (Kipf and Welling, 2016):

(2) $$q(Z|F,E)=\prod_{i=1}^{N}q(z_{i}|F,E),\;\mathrm{where}\;q(z_{i}|F,E)=N(z_{i}|\mu_{i},\sigma_{i}^{2}),$$

where $\mu =GCN_{\mu }(F,E)$ is the mean of the latent vectors, while *μ_i_* is the *i*-th element of it, which is inferred by GCN to model $q(z_{i}|F,E)$ as a Gaussian distribution. Similarly, $\sigma =GCN_{\sigma }(F,E)$ is the standard deviation of the latent vector that is inferred by another GCN, and *σ_i_* is the *i*-th element of it. *Z* can be sampled from the distributions of the latent vectors q(Z|F,E).

The edge and node attributes are inputted into the convolution layer, which is based on the classical GCN (Kipf and Welling, 2016). During convolution, for each graph, given the node attribute matrix *F* and the edge attribute matrix *E*, we then have $H=E^{\prime }FW$, where *W* is a matrix that refers to a layer-specific trainable weight matrix and $E^{\prime }=D^{-1/2}ED^{-1/2}$. *D* refers to the normalized degree matrix of the graph. More details on the operations in a GCN can be found in Kipf and Welling (2016).

For the generator to approximate p(E,F|Z), we utilize the graph decoder developed in our prior works (Guo *et al.*, 2018) based on novel graph deconvolution operations for graph decoding. In our problem of contact graph generation, the node attribute matrix is fixed. Thus, we can first generate *F* by directly copying from the input. To generate the edge attribute *E*, the latent vector *Z* is first inputted into a fully connected layer to get Z′. Then, Z′ is concatenated with each node feature vector in *F* to get the new node feature matrix F′. F′ is inputted into the graph deconvolution network, including node deconvolution layers and edge deconvolution layers, to obtain the final generated edge matrix *E*.

We perform mini-batch gradient descent and make use of the re-parameterization trick (Kingma and Welling, 2014) to optimize the parameters of the Gaussian distribution.

### 2.3 Disentanglement enhancement contact graph VAE

Although disentanglement enhancement and deep graph generative models are respectively attracting increasing attention in recent years, their integration remains under-explored (Higgins *et al.*, 2017; You *et al.*, 2018). The DECO-VAE model, which stands for Disentanglement Enhancement Contact Graph Variational Autoencoder, represents one of the few works to integrate the two and the first work to do so for protein structure modeling. Specifically, we propose to generalize the CO-VAE framework with an additional hyperparameter *β* that modulates and enhances the independency among the individual latent variables of *Z*; the disentanglement enhancement mechanism is inspired by *β*-VAE (Higgins *et al.*, 2017). The goal is to weaken the coupling among the latent variables. Equation (1) can be re-written to obtain the DECO-VAE formulation, with the addition of the *β* coefficient:

(3) $$\ell =E_{q(Z|F,E)}[\text{log}\;p(E,F|Z)]-\beta KL[q(Z|F,E)||p(Z)].$$

When *β* = 1, DECO-VAE becomes equivalent to CO-VAE. When β>1, the model is pushed to pursue more toward the consistency with the isotopic Gaussian prior, thereby enhancing variable independency.

### 2.4 Details of architecture and training parameters of CO-VAE and DECO-VAE

We describe the detailed operation and implementation of the encoder and decoder in the CO-VAE and DECO-VAE frameworks. The training parameters and architecture information are also provided for reproducibility. The encoder, which is the same both in CO-VAE and DECO-VAE, consists of two edge convolution layers [proposed by Guo *et al.* (2018)] sequentially and then two paths of graph convolution layers [proposed in Kipf and Welling (2017)] for inferring the latent representation *Z*, which follows the Gaussian distribution. One path is used to generate the mean of Gaussian distribution, and another path is used to generate the standard derivation. The decoder consists of one fully connected layer, a node deconvolution layer [proposed in Guo *et al.* (2018)] and two edge deconvolution layers [proposed by Guo *et al.* (2018)]. We summarize the parameters of the architecture and training process in the Supplementary Material. The node and edge convolution layer is described in the format of < Filter_size > < Conv_type >. < Num_Channels > < Activationfunction > .stride(*s*) < stride_size >. The graph convolution layer is described in the format of: Graph − conv. < Num_Channels > < Activationfunction >. The fully connected layer is described as: FC: < Num_of_hiddens >. The learning rate for training is $5\times 10^{-4}$; the mini-batch is 100, and the number of epochs is 200.

Implementation Details: codes for the neural network layers of decoder can be found at https://github.com/anonymous1025/Deep-Graph-Translation-, and codes for layers of encoder can be found at https://github.com/tkipf/gcn.

## 3 Results

### 3.1 Experimental setup

#### 3.1.1 Datasets

The evaluation is carried out on 15 protein targets of varying lengths (53 to 146 amino acids long). Column 2 in Table 1 lists the name of each protein; Column 3 reports the length in the number of amino acids and Column 4 relates the number of tertiary structures generated per protein with the Rosetta AbInitio protocol (Leaver-Fay *et al.*, 2011). Specifically, on each protein target, using as input its amino acid sequence in fasta format, the Rosetta AbInitio protocol [available freely in the Rosetta software suite (Leaver-Fay *et al.*, 2011)] is run in an embarrassingly parallel manner to obtain a set of at least 50 000 tertiary structures. The same amount of time is allocated per protein; the variation in the number of tertiary structures obtained is due to the variation in the cost of energy calculations in Rosetta as a function of chain length. The resulting structures are converted into contact graphs as described in Section 1. A 4:1 split is used to obtain the training and testing dataset on each protein.

**Table 1.** Protein targets employed for evaluation

	Protein	Num_AA	Dataset size
1.	Rubredoxin (formyl methionine mutant) from *Pyrococcus furiosus*	53	61 000
2.	Human carboxypeptidase A2	61	58 745
3.	High-potential iron-sulfur protein	62	60 000
4.	Chromosomal protein SSO7D	64	65 000
5.	B1 domain of protein L	64	60 000
6.	Hyperthermophile protein	66	66 000
7.	Chey-binding domain of Chea	69	51 724
8.	N-terminal fragment of NS1 protein from influenza A	70	58 491
9.	Nova-2 KH3 K-homology RNA-binding domain	74	60 500
10.	N-terminal domain of *Escherichia coli* arginine repressor	78	57 000
11.	Azobacter cytochrome C5	83	55 000
12.	Translation initiation factor 3 C-terminal domain	88	60 000
13.	Aspartyl protease from HIV-1 isolate BRU	99	60 000
14.	PG1108 from *Porphyromonas gingivalis* W83	123	54 795
15.	Extracellular fragment of human CD40	146	53 000

*Note*: Column 3 shows the number of amino acids (Num_AA) in a protein target. The number of tertiary structures (dataset size) in the Rosetta-generated dataset for each protein target is shown in Column 4.

### 3.2 Comparison with SOTA methods

We compare the proposed CO-VAE and DECO-VAE to four baseline methods, VGAE, Graphite, GraphRNN and WGAN (described in detail in the Supplementary Material). In summary, Graphite (Grover *et al.*, 2019) is a framework for unsupervised learning of representations over nodes in large graphs using deep latent variable generative models. VGAE (Kipf and Welling, 2016) is implemented based on the VAE framework for unsupervised learning on graph-structured data. GraphRNN (You *et al.*, 2018) is a deep autoregressive model to approximate any distribution of graphs with minimal assumptions about their structure. WGAN (Rahman *et al.*, 2021) is a GAN that leverages image convolution and Wasserstein distance in the loss function to generate distance matrix representations of tertiary protein structures.

**Fig. 2.**
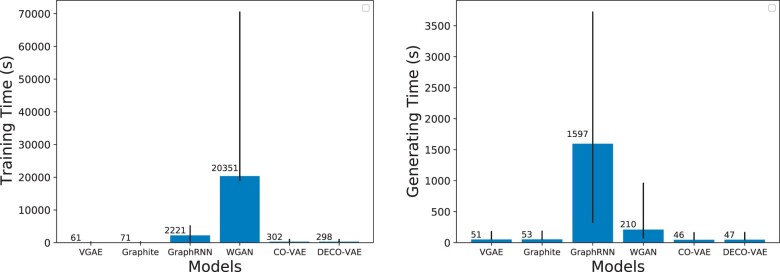
Average (left) training and (right) generating times for the models under comparison. The error bars indicate the range

Our first comparison focuses on training and generating times, which vary greatly among the models. For instance, the left panel of Figure 2 shows training times varying from 61.06 s for VGAE, to 71.5 s for Graphite, to 298.06 s for DECO-VAE, to 302.43 s for CO-VAE, to 2221.63s for GraphRNN and to 20 351s for WGAN. The left panel of Figure 2 also indicates that the largest range (indicated by vertical lines) is observed for GraphRNN and then WGAN, in this order. The right panel of Figure 2 shows a similar pattern, with CO-VAE and DECO-VAE having the lowest average generating times, followed closely by VGAE and Graphite. GraphRNN has the highest average (and range of) generating time, followed next by WGAN.

### 3.3 Evaluating the quality of the generated datasets

Our first comparison focuses on evaluating the quality of the generated datasets, using the training dataset as a reference. Specifically, we evaluate whether the generated contact graphs from the learned distribution follow that of the training samples. We do so in two ways, first by focusing on intrinsic graph properties and so summarizing a contact graph with a summary statistic (such as density, or number of edges, etc., as described below), or by focusing on domain-specific metrics that additionally leverage experimentally known structures.

### 3.4 Evaluating learned distributions via graph-intrinsic properties

First, we focus on four intrinsic graph properties: the *density* of a graph, the *number of edges* in it, the *average degree coefficient* and the *transitivity* of a graph. The density of a graph is the ratio between the number of edges in the graph and the number of possible edges (that would be achieved in a complete graph with the same number of vertices). The average degree coefficient measures the similarity of connections in the graph with respect to the node degree. Transitivity is the overall probability for the graph to have adjacent nodes interconnected. We calculate these properties via the open source API NetworkX (Hagberg *et al.*, 2008).

The distance between the distribution of the generated contact graphs and the distribution of the graphs in the training dataset in terms of each of the four properties is measured in three ways: via the Pearson correlation coefficient (PCC) (Benesty *et al.*, 2009), the Bhattacharyya distance (BD) (Kailath, 1967) and earth mover’s distance (EMD) (Rubner *et al.*, 2000). In statistics, PCC measures the linear correlation between two variables *X* and *Y*. Here, *X* and *Y* refer to a specific graph property measured over the generated versus the training graphs, respectively. BD and EMD both measure the dissimilarity/distance of two probability distributions.

**Fig. 3.**
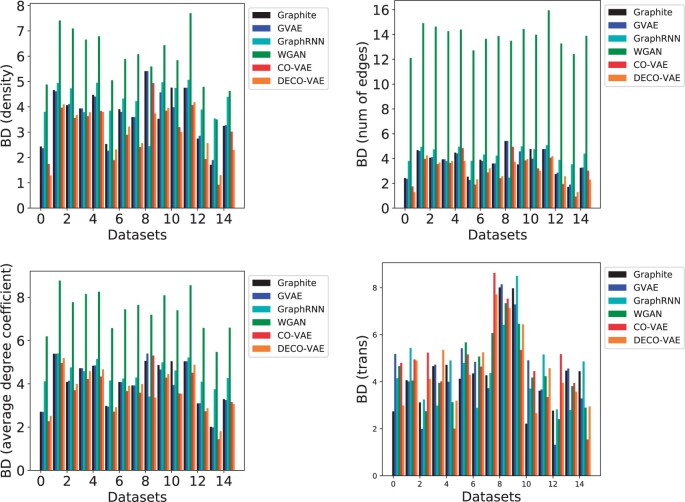
The generated graphs are compared to the ones in the training dataset on four graph properties. BD is used to measure the distance between two distributions. The boxplots show the range of BD values over all compared models across all datasets. Annotations denote the values obtained by CO-VAE and DECO-VAE

In the interest of space, we only relate here in Figure 3 the BD-based comparison along each of the four graph properties. All results are shown in the Supplementary Material. Bar plots are used to compare the various models.

Figure 3 shows that the lowest BDs are obtained either by CO-VAE or DECO-VAE; i.e. the generated distribution is most similar to the distribution in the training dataset (according to the four graph properties and according to BD) when the distribution is generated with either of the two models proposed here. The worst BD is obtained by WGAN. In the Supplementary Material, a new view of the BD-based comparison that does not include WGAN is shown. Figure 3 shows that the lowest BD is achieved by CO-VAE on all of the four properties with an average value of 3.02; DECO-VAE reports the second lowest BD value of the four properties with an average of 3.26, which is very similar to that reported by CO-VAE (see Supplementary Material for all values in table format). The Supplementary Material additionally shows that the lowest EMD is almost always achieved by CO-VAE or DECO-VAE on each of the four graph properties.

### 3.5 Evaluating learned distributions via biomolecular domain-specific metrics

Alternatively, experimentally available structures can be used as a reference. Specifically, for each protein, we obtain an experimentally available structure from the Protein Data Bank (Berman *et al.*, 2003) (listed in the Supplementary Material), convert it into a contact graph and count the number of edges in it; we refer to these as ‘native contacts’. Specifically, we introduce two metrics, NAT-C and NONNNAT-C, to summarize a generated contact graph.

NAT-C measures the percentage of native contacts in a contact graph. Given a contact graph, the number of edges (contacts) that are also found in a given native contact graph is divided by the total number of edges in the native contact graph. This ratio is turned into a percentage. In the summary analysis below, we report on the average of NAT-Cs measured over the contact graphs in a dataset.

NONNAT-C reports on the non-native contacts; i.e. the edges in a contact graph that are not found in the native contact graph. Since there is no reasonable way to normalize the number of these edges, we measure NONNAT-C somewhat differently. Specifically, given a contact graph, we first measure the number of edges in it that are not found in a given native contact graph (so, the number of non-native contacts). This number is normalized by the number of amino acids (number of vertices). In this way, NONNAT-C reports on the number of non-native contacts per amino acid. We report on the average of NONNAT-Cs measured over the contact graphs in a dataset.

**Fig. 4.**
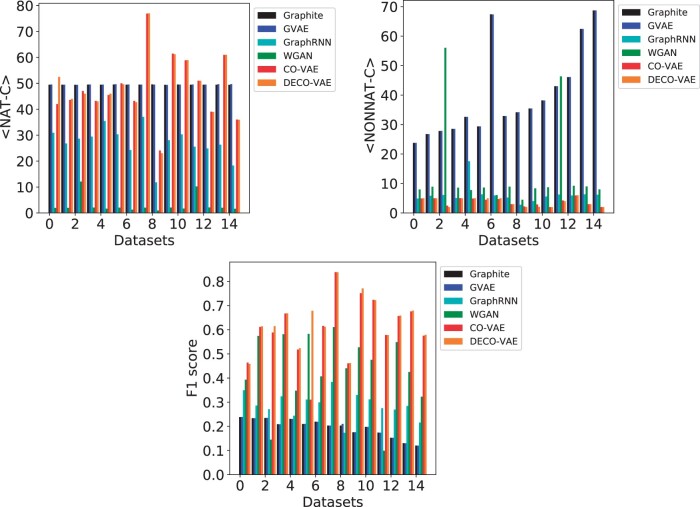
The generated graphs are compared to a reference graph computed over an experimentally-available structure for each protein; <NAT-C>, <NONNAT-C> and *F*1 scores are computed over generated graphs. The bar plots show the values obtained from each model across all datasets

The top panel of Figure 4 shows <NAT-C>, the average NAT-C and <NONNAT-C>, the average NONNAT-C, over generated contact graphs for each of the five models; Supplementary Table S3 lists all the values in table format.

The top panel of Supplementary Figure S4 and Table S3 shows that the highest <NAT-C> is achieved by Graphite on 8/15 of the protein targets, followed by DECO-VAE and CO-VAE, each of which achieves the highest <NAT-C> values on three protein targets, and then VGAE, which achieves the highest on one target. While GraphRNN achieves the lowest <NAT-C> values over all protein targets, DECO-VAE and CO-VAE achieve no lower than 7% of the highest <NAT-C> achieved by Graphite on 6/8 targets where Graphite outperforms other models. VGAE and Graphite report <NONNAT-C> values almost an order of magnitude higher than those achieved by GraphRNN, CO-VAE and DECO-VAE, which points to unrealistic tertiary structures. In fact, CO-VAE or DECO-VAE achieve the lowest <NONNAT-C> values on 12/15 targets. The only two models that achieve both high <NAT-C> and low <NONNAT-C> values are CO-VAE and DECO-VAE. These results suggest that CO-VAE and DECO-VAE generate (physically realistic) contact graphs with high NAT-C and low NONNAT-C values compared to the other three models. These results are supported visually in Supplementary Figure S3 on three selected target proteins, where the distribution of NAT-C and NONNAT-C values over the training dataset are compared visually to the corresponding distributions over the data generated by DECO-VAE.

### 3.6 Precision, recall, coverage and F1 score on biomolecular domain-specific metrics

Using the contact graph of a reference experimental structure for each protein as the ‘ground truth’, we calculate the precision, recall, coverage and *F*1 score over the generated dataset for each of the five models under comparison. In summary, we recall that *precision* = true positive (TP)/[(TP + false positive (FP)]. We interpret an edge in a generated contact graph that is also in the native contact graph as a TP. Conversely, an edge in a generated contact graph that cannot be found in the native contact graph is an FP. We also note that *recall* = TP/[(TP + false negative (FN)], where an edge that can be found in the native contact graph but is missing in a generated contact graph is counted as an FN. The coverage metric measures only the number of TPs. The *F*1 score combines both precision and recall as in *F1* = 2 × Recall × Precision/(Recall + Precision). The use of the harmonic mean makes the *F*1 score more sensitive to the smaller of the two values.

The bottom panel of Figure 4 highlights the *F*1 scores; the Supplementary Material shows all these values in table format. CO-VAE and DECO-VAE achieve very high *F*1 scores in the range of 0.61 and 0.64 on average, which is over 52.4% and 54.7% higher than the best-performing baseline model (among the three other models). The Supplementary Material additionally shows that CO-VAE and DECO-VAE additionally achieve high coverage and recall of around 0.34, outperforming the other models by a large margin. The precision reached by CO-VAE and DECO-VAE is in the range of 0.67 and 0.71 on average, which is over 53.4% and 56.7% higher than the best-performing baseline model. Similarly, the coverage reached by CO-VAE and DECO-VAE is in the range of 0.55 and 0.58 on average, which is over 12.7% and 24.8% higher than the best-performing baseline model. The recall reached by CO-VAE and DECO-VAE is in the range of 0.57 and 0.59 on average, which is over 12.2% and 15.2% higher than the best-performing baseline model. In summary, CO-VAE and DECO-VAE, obtain the best performance in all four metrics on almost 76% of the target proteins.

### 3.7 Comparing generated distributions to the training dataset

The Supplementary Material additionally compares the CO-VAE- and DECO-VAE-generated datasets to the training dataset on each target, utilizing the <NAT-C> and <NONNAT-C> metrics. On each target, the <NAT-C> or <NONNAT-C> values, respectively, on the training dataset are used as a reference, and the *δ* improvement in the respective metric in the generated dataset is measured and reported. Supplementary Figure S5 shows that the contact graphs generated by CO-VAE and DECO-VAE are of high quality and resemble the training datasets in native and non-native contacts across the tertiary structures. The lowest differences in the average percentage of native contacts (over the generated dataset from a reference training dataset) are reached by CO-VAE on 6/15 of the targets and by DECO-VAE on 10/15 on the targets (with ties in one target). The lowest differences in the average number of non-native contacts per amino acid are reached by CO-VAE on 12/15 of the targets and by DECO-VAE on 7/15 of the targets (with ties in 4/15 of the targets).

**Fig. 5.**
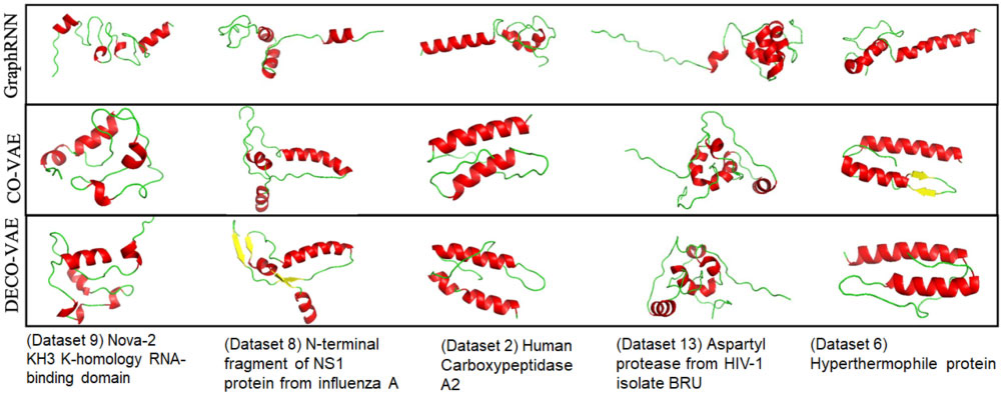
Some generated structures are shown for five selected proteins. A generated contact graph is converted via CONFOLD into a tertiary structure, which is drawn with PyMol (DeLano, 2002). Secondary structures are shown (alpha helices in red, beta sheets in yellow and coils in green)

The evaluation so far shows that CO-VAE and DECO-VAE produce more protein-like contact graphs. We provide some representative tertiary structures, reconstructed with CONFOLD (Adhikari *et al.*, 2015) from generated contact graphs, for CO-VAE, DECO-VAE and GraphRNN on each of several selected proteins in Figure 5. The structures shown in Figure 5 are physically realistic, with secondary structures (alpha helices, beta sheets and coils) packed around one another. Qualitatively, there is more secondary structure and better packing in CO-VAE and DECO-VAE.

### 3.8 Interpreting the disentangled latent representation learned in DECO-VAE

**Fig. 6.**
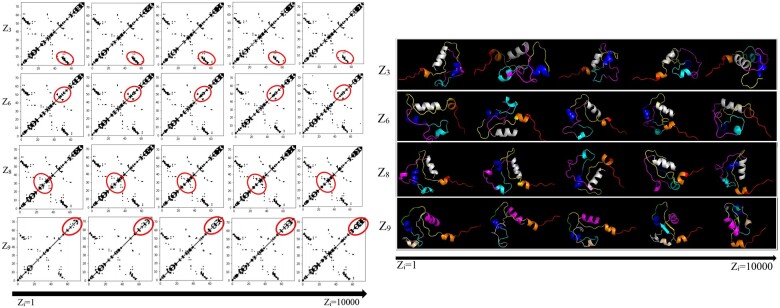
Left: generated contact graphs for a selected protein target; four semantic factors in the latent variables (i.e. *Z*_3_, *Z*_6_, *Z*_8_ and *Z*_9_) control changes in the contact graphs; the value of latent variables changes from 1 to 10 000; right: corresponding reconstructed tertiary structures

In addition to being one of the top-performing methods on the various evaluations above, DECO-VAE learns a disentangled representation. By changing the value of one variable in the latent code continuously and fixing the remaining variables, we can visualize the corresponding change of the generated contact graph. We do so for a selected protein target, the NOVA-2 KH3 K-homology RNA-binding domain. The left panel in Figure 6 shows the change in contact maps, and the right panel shows the corresponding tertiary structures [reconstructed with CONFOLD (Adhikari *et al.*, 2015)]. A latent code varies from left to right; when a latent code varies, the others and noise are fixed. Each row in Figure 6 corresponds to each individual varying factor (while fixing the others) that is controlled by one variable in the learnt latent codes. For example, the first row shows five generated contact maps for five possible values (ranging from 1 to 10 000) of latent variable *Z*_3_. From the varying contact maps in each row, one can see which part is related to or controlled by the corresponding latent factor/variable. We highlight some parts (red circles) that are consistently influenced by each factor. What may seem like small changes in the contact map translate to global rearrangements of secondary structures couple with local unfolding and folding of the secondary structures. While in other domains, such as vision, it is often the case that the latent factors control disparate, easily perceptible information, such as color, texture and so on, in domains, such as molecular structure modeling, the information encoded in the latent factors is not easily perceptible. This is the reason we relate via this analysis both changes in contact maps and corresponding tertiary structures. Similar observations and analyses on other proteins are related in the Supplementary Material.

## 4 Conclusion

Currently, popular methods for exploring protein structure spaces build upon the Monte Carlo or Molecular Dynamics. One practical appeal of the generative models, we propose here is their low-computational times in generating structures. For instance, CO-VAE and DECO-VAE spend less than a millisecond in generating one contact graph (considering training time as pre-processing cost). In contrast, it takes anywhere from several seconds to a minute (or more) to generate a tertiary structure with a Metropolis Monte Carlo method.

Altogether, the presented results are encouraging and the first step toward maturing generative models. As we summarize in Section 1, much of the work on generating physically realistic tertiary structures leverages GANs rather than VAEs. GANs are easier to implement, but they are also more difficult to train properly, and suffer from well-known issues. As reported in Rahman *et al.* (2021), these issues need to be diagnosed and addressed, lest the quality of generated structures suffers. In addition, as the evaluation in Rahman *et al.* (2021), GANs seem to have a hard time learning all the rich information in a tertiary structure, such as the presence of a backbone, the short-range contacts that indicate the secondary structures, and the long-range contacts that indicate the packing of those secondary structures in three dimensions. The generative network in a GAN has a hard time balancing all these constraints, and so much work focuses on heuristics to remedy one issue at a time. In contrast, we do not observe such difficulties in VAEs. Most centrally, this is due to the embedding in contact graph space. All current GAN-based work utilizes convolution (as in images). Though in a controlled setting, learning over rich datasets generated by reliable models over various protein targets, the graph-based VAE methods presented here show much promise.

Encouraged by these results, there is much future research, we hope to investigate. For instance, the exploration of the node-edge joint generative model would be an interesting direction. The proposed DECO-VAE focuses on generating the graph topology (i.e. via contacts) instead of node features (e.g. properties and types of amino acids). Jointly generating both graph topology and node features could be important to further improve the quality of the generated distributions. A differentiable model that generates tertiary structures instead of relying on contact-to-tertiary structure reconstruction methods would also be beneficial to provide a direct interpretation of what the latent variables control and perhaps a further insight into improving the quality of the generated datasets. This direction can additionally leverage pairwise amino acid distances instead of contact graphs.

Another direction to explore is not to rely on training datasets obtained for specific sequences (as we do here) but instead to directly utilize known experimental structures. This is non-trivial, as such structures are obtained for different amino acid sequences and thus have different lengths and characteristics. However, such a direction may prove useful to further diversify generated structures and potentially explore a larger latent space containing novel structures currently beyond the reach of the wet laboratory. We also note that due to the setup pursued in this article, the generated structures are sequence specific (as the training dataset is sequence-specific). When moving forward with generative frameworks that can leverage experimentally available structures of varying protein sequences, an important computational component will be to condition generated structures upon a specific sequence.

## Author contributions

X.G., Y.D., L.Z. and A.S. conceived the methodology and the experiment(s), X.G., Y.D. and S.T. conducted the experiment(s), X.G., Y.D. and S.T. analyzed the results. X.G., Y.D., L.Z. and A.S. wrote and reviewed the manuscript.

## Funding

This work was supported in part by funds from the National Science Foundation (NSF: # 1942594, # 1755850 and # 1907805). This material is additionally based upon work by A.S. supported while serving at the National Science Foundation.

*Conflict of Interest*: none declared.

## Supplementary Material

vbab036_Supplementary_Data

## References

[vbab036-B1] Adhikari B. et al (2015) CONFOLD: residue-residue contact-guided ab initio protein folding. Proteins, 83, 1436–1449. 25974172 10.1002/prot.24829 PMC4509844

[vbab036-B2] Benesty J. et al (2009) Pearson correlation coefficient. In: Noise Reduction in Speech Processing. Vol 2, Springer, Berlin, Heidelberg, pp. 1–4. 10.1007/978-3-642-00296-0_5.

[vbab036-B3] Berman H.M. et al (2003) Announcing the worldwide Protein Data Bank. Nat. Struct. Biol., 10, 980. 14634627 10.1038/nsb1203-980

[vbab036-B4] Boehr D.D. , Wright P.E. (2008) How do proteins interact? Science, 320, 1429–1430. 18556537 10.1126/science.1158818

[vbab036-B5] Boehr D.D. et al (2009) The role of dynamic conformational ensembles in biomolecular recognition. Nat. Chem. Biol., 5, 789–796. 19841628 10.1038/nchembio.232 PMC2916928

[vbab036-B6] DeLano W.L. (2002) The PyMOL Molecular Graphics System. Delano Scientific, San Carlos.

[vbab036-B7] Ding W. , Gong H. (2020) Predicting the real-valued inter-residue distances for proteins. Adv. Sci., 7, 2001314. 10.1002/advs.202001314 PMC7539185 33042750

[vbab036-B8] Grover A. et al (2019) Graphite: iterative generative modeling of graphs. Int. J. Mach. Learn. Res., 80, 1–11.

[vbab036-B9] Guo X. et al (2018) Deep graph translation. *arXiv preprint arXiv:1805.09980*.

[vbab036-B10] Hagberg A. et al (2008) Exploring network structure, dynamics, and function using networkx. *Technical report*. Los Alamos National Lab (LANL), Los Alamos, NM, USA.

[vbab036-B11] Henderson R. et al (2020) Controlling the SARS-CoV-2 spike glycoprotein conformation. Nat. Struct. Mol. Biol., 27, 925–933. 32699321 10.1038/s41594-020-0479-4 PMC8581954

[vbab036-B12] Higgins I. et al (2017) beta-VAE: learning basic visual concepts with a constrained variational framework. In: *International Conference on Representation Learning*, pp. 1–22. Toulon, France.

[vbab036-B13] Hoseini P. et al (2021) Generative deep learning for macromolecular structure and dynamics. Curr. Opin. Struct. Biol., 67, 170–177. 33338762 10.1016/j.sbi.2020.11.012

[vbab036-B14] Jumper J. et al (2021) Highly accurate protein structure prediction with AlphaFold. Nature, 596, 583–589. 34265844 10.1038/s41586-021-03819-2 PMC8371605

[vbab036-B15] Kailath T. (1967) The divergence and Bhattacharyya distance measures in signal selection. IEEE Trans. Commun. Technol., 15, 52–60.

[vbab036-B16] Kingma D.P. , Welling M. (2014) Auto-encoding variational Bayes. In: *International Conference on Representation Learning*, pp. 1–14. Banff, Canada.

[vbab036-B17] Kipf T.N. , Welling M. (2016) Variational graph auto-encoders. *arXiv preprint arXiv:1611.07308*.

[vbab036-B18] Kipf T.N. , Welling M. (2017) Semi-supervised classification with graph convolutional networks. In: *International Conference on Representation Learning*. pp. 1–14. Toulon, France.

[vbab036-B19] Kullback S. (1997) Information Theory and Statistics. Dover Publications, New York.

[vbab036-B20] Leaver-Fay A. et al (2011) ROSETTA3: an object-oriented software suite for the simulation and design of macromolecules. Methods Enzymol., 487, 545–574. 21187238 10.1016/B978-0-12-381270-4.00019-6 PMC4083816

[vbab036-B21] Majumder S. et al (2021) Exploring the intrinsic dynamics f SARS-CoV-2, SARS-CoV and MERS-CoV spike glycoprotein through normal mode analysis using anisotropic network model. J. Mol. Graph. Model., 102, 107778. 33099199 10.1016/j.jmgm.2020.107778 PMC7567490

[vbab036-B22] Maximova T. et al (2016) Principles and overview of sampling methods for modeling macromolecular structure and dynamics. PLoS Comput. Biol., 12, e1004619. 27124275 10.1371/journal.pcbi.1004619 PMC4849799

[vbab036-B23] Nussinov R. et al (2019) Computational structural biology: the challenges ahead. Molecules, 24, 637. 30759724 10.3390/molecules24030637 PMC6384756

[vbab036-B24] Rahman T. et al (2021) Generative adversarial learning of protein tertiary structures. Molecules, 26, 1209. 33668217 10.3390/molecules26051209 PMC7956369

[vbab036-B25] Rubner Y. et al (2000) The earth mover’s distance as a metric for image retrieval. Int. J. Comput. Vis., 40, 99–121.

[vbab036-B26] Tian H. , Tao P. (2021) Deciphering the protein motion of S1 subunit in SARS-CoV-2 spike glycoprotein through integrated computational methods. J. Biomol. Struct. Dyn., 39, 6705–6712. 32746720 10.1080/07391102.2020.1802338 PMC7484573

[vbab036-B27] Vendruscolo M. et al (1997) Recovery of protein structure from contact maps. Fold. Des., 2, 295–306. 9377713 10.1016/S1359-0278(97)00041-2

[vbab036-B28] Yang H. et al (2020) GANCon: protein contact map prediction with deep generative adversarial network. IEEE Access, 8, 80899–80907.

[vbab036-B29] You J. et al (2018) GraphRNN: generating realistic graphs with deep auto-regressive models. Int. J. Mach. Learn. Res., 80, 1–10.

