# Supplementary material for: Generating tertiary protein structures via interpretable graph variational autoencoders
Source: Bioinform Adv. 2021 Nov 29;1(1):vbab036. doi: 10.1093/bioadv/vbab036 (PMC9710582; doi:10.1093/bioadv/vbab036)
Supplement: vbab036_Supplementary_Data [file vbab036_supplementary_data.pdf]

Supplementary Data

Architecture and Training Parameters

We describe the detailed operation and implementation of the encoder and decoder in the comparison methods: VGAE, Graphite and GraphRNN. VGAE parameterizes VAEs with graph neural networks and uses a novel iterative graph refinement strategy inspired by low-rank approximations for decoding. GraphRNN learns to generate graphs by training on a representative set of graphs and decomposes the graph generation process into a sequence of node and edge formations, conditioned on the graph structure generated so far. We summarize the training parameters and architecture information of VGAE and Graphite for reproducibility below. The neural network layer is described in the format of $< LayerType > . < Num\_hiddens >$. The GraphRNN model is the same as that provided in arXiv preprint arXiv:1802.08773. The WGAN model is the same as the one provided in (Rahman et al. 2021). The learning rate to train the deep neural network model for all the comparison models is 5×10^{-4}; the mini-batch is 100 and the number of epochs is 200. Codes for all the utilized neural network layers can be found at: `https://github.com/tkipf/gae` (VGAE), `https://github.com/ermongroup/graphite` (Graphite), and `https://github.com/JiaxuanYou/graph-generation` (GraphRNN).

Table 1. Training parameters, encoder, and decoder architecture. N refers to the number of amino acids.

VGAE

Graph encoder	Graph decoder
Input: $F \in \mathbb{R}^{N\times 16}$, $A \in \mathbb{R}^{N\times N}$	Input: $z \in \mathbb{R}^{N\times 20}$
Graph-conv.10 ReLU	InnerProduct Layer
Graph-conv.10 ReLU	

Graphite

Graph encoder	Graph decoder
Input: $F \in \mathbb{R}^{N\times 16}$, $A \in \mathbb{R}^{N\times N}$	Input: $z \in \mathbb{R}^{N\times 20}$
Graph-conv.10 ReLU	Graphite Layer.10
Graph-conv.10 ReLU	InnerProduct Layer

CO-VAE & DECO-VAE

Graph encoder	Graph decoder
Input: $F \in \mathbb{R}^{N\times 16}$, $A \in \mathbb{R}^{N\times N}$	Input: $z \in \mathbb{R}^{20}$
2×2 Edge-conv.10 ReLU. s. 1	FC.20 $\cdot N$
2×2 Edge-conv.5 ReLU. s. 1	$N \times 1$ Node-deconv.4 ReLU. s. 1
Graph-conv.4 ReLU	$N \times 1$ Edge-deconv.5 ReLU. s. 1
FC.20	20×1 Edge-deconv.10 ReLU. s. 1

Further Evaluation

Supplementary Fig. 1 compares the five models excluding WGAN across the four intrinsic graph properties described in the main manuscript. Supplementary Table 2 provides the actual values and includes the PCC- and EMD-based comparison.

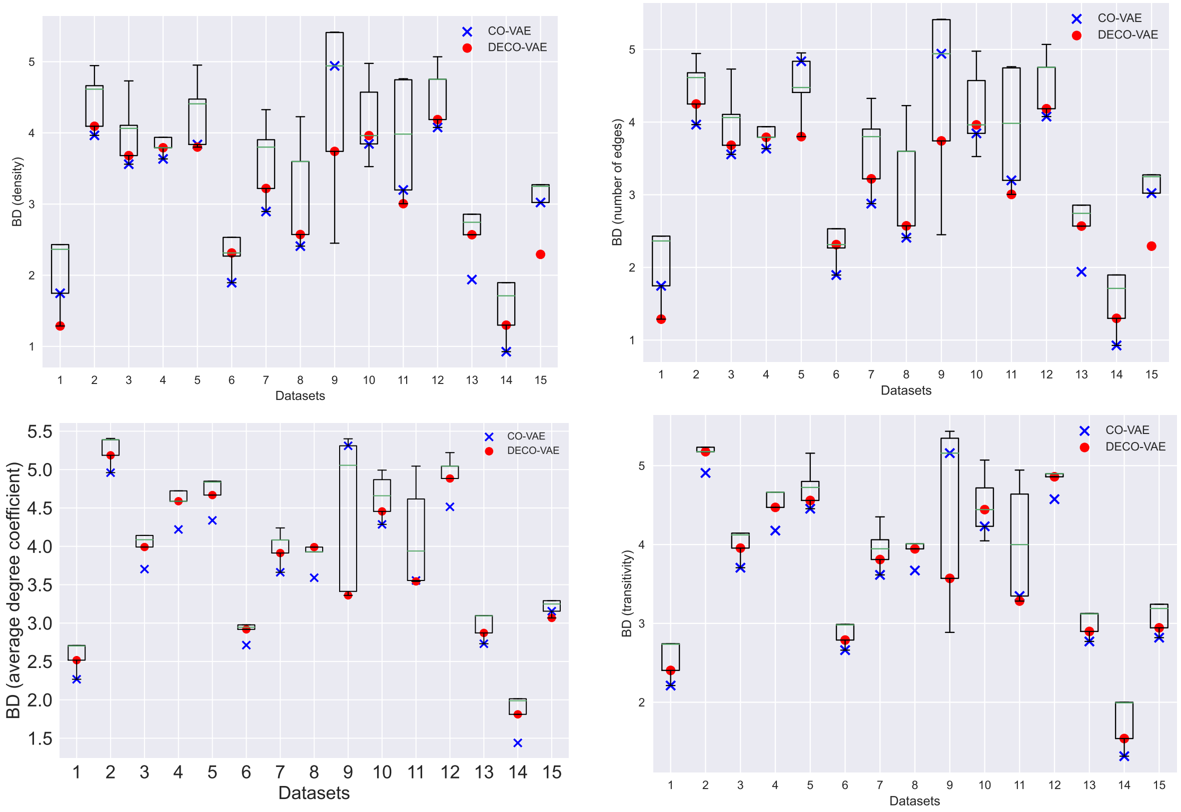

Fig. 1. Generated graphs are compared to the ones in the training dataset on four graph properties. BD measures the distance between distributions. Boxplots show the range of BD values over five models. Annotations denote CO-VAE and DECO-VAE.

Table 2. Comparison of learned distributions (on each of the four graph properties of interest, listed in the rows) by their distance (according to PCC, BD, and EMD) to the training dataset for different proteins. The lowest BD and EMD values on each of the four graph properties of interest are highlighted in bold font.

	Graph Property	Methods	PCC	BD	EMD
D 1	Density	Graphite	**0.062**	2.431	0.384
		GVAE	0.023	2.364	0.384
		GraphRNN	0.053	3.801	0.013
		WGAN	0.000	4.880	0.004
		CO-VAE	0.008	1.747	**0.002**
		DECO-VAE	0.051	**1.287**	**0.002**
	N Edges	Graphite	**0.062**	2.431	549.203
		GVAE	0.023	2.364	549.940
		GraphRNN	0.053	3.801	18.021
		WGAN	0.000	12.108	5.931
		CO-VAE	0.008	1.747	**2.278**
		DECO-VAE	0.051	**1.288**	2.290
	Avg-Deg-Cor	Graphite	0.014	2.709	0.045
		GVAE	0.040	2.702	0.047
		GraphRNN	0.022	4.116	0.127
		WGAN	0.000	6.194	0.026
		CO-VAE	**0.059**	2.268	0.017
		DECO-VAE	0.016	**2.517**	**0.010**
	Transitivity	Graphite	0.001	2.736	0.090
		GVAE	0.022	2.742	0.090
		GraphRNN	0.063	4.124	0.240
		WGAN	0.000	6.069	0.047
		CO-VAE	**0.069**	**2.212**	**0.003**
		DECO-VAE	0.023	2.404	0.004

	Graph Property	Methods	PCC	BD	EMD
D 2	Density	Graphite	0.010	4.663	0.386
		GVAE	0.000	4.614	0.386
		GraphRNN	0.011	4.945	0.009
		WGAN	0.000	7.410	0.002
		CO-VAE	**0.018**	**3.965**	0.001
		DECO-VAE	0.010	4.093	**0.000**
	N Edges	Graphite	0.010	4.679	706.323
		GVAE	0.000	4.613	706.720
		GraphRNN	0.011	4.944	16.698
		WGAN	0.000	14.922	4.260
		CO-VAE	**0.018**	**3.965**	1.703
		DECO-VAE	0.010	4.249	**0.801**
	Avg-Deg-Cor	Graphite	0.008	5.389	0.090
		GVAE	0.020	5.389	0.089
		GraphRNN	0.003	5.406	0.149
		WGAN	0.000	8.772	0.011
		CO-VAE	**0.024**	**4.960**	**0.003**
		DECO-VAE	0.007	5.186	0.007
	Transitivity	Graphite	0.003	5.178	0.093
		GVAE	0.019	5.234	0.093
		GraphRNN	0.006	5.421	0.254
		WGAN	0.000	8.630	0.035
		CO-VAE	0.003	**4.908**	**0.002**
		DECO-VAE	**0.012**	5.177	**0.002**

	Graph Property	Methods	PCC	BD	EMD
D 3	Density	Graphite	**0.015**	4.063	0.401
		GVAE	0.011	4.106	0.400
		GraphRNN	0.004	4.730	**0.003**
		WGAN	0.000	7.094	0.781
		CO-VAE	0.007	**3.560**	0.036
		DECO-VAE	0.011	3.681	0.038
	N Edges	Graphite	**0.015**	4.063	758.513
		GVAE	0.011	4.106	756.483
		GraphRNN	0.004	4.730	**5.640**
		WGAN	0.000	14.639	1477.466
		CO-VAE	0.007	**3.556**	67.921
		DECO-VAE	0.011	3.681	72.522
	Avg-Deg-Cor	Graphite	0.009	4.086	0.131
		GVAE	0.008	4.142	0.131
		GraphRNN	0.006	4.760	0.179
		WGAN	0.000	7.776	0.346
		CO-VAE	0.027	**3.703**	**0.036**
		DECO-VAE	**0.040**	3.991	0.063
	Transitivity	Graphite	0.021	4.145	0.089
		GVAE	0.017	4.122	0.089
		GraphRNN	0.005	4.799	0.292
		WGAN	0.000	7.712	0.399
		CO-VAE	0.000	**3.706**	**0.049**
		DECO-VAE	**0.034**	3.955	0.050

	Graph Property	Methods	PCC	BD	EMD
D 4	Density	Graphite	0.011	3.937	0.392
		GVAE	0.011	3.937	0.392
		GraphRNN	0.023	3.791	0.003
		WGAN	0.000	6.658	0.008
		CO-VAE	0.021	**3.634**	**0.001**
		DECO-VAE	**0.024**	3.791	**0.001**
	N Edges	Graphite	0.011	3.937	789.502
		GVAE	0.011	3.937	789.500
		GraphRNN	0.023	3.791	1.286
		WGAN	0.000	14.267	15.833
		CO-VAE	0.021	**3.634**	3.015
		DECO-VAE	**0.024**	3.791	**1.187**
	Avg-Deg-Cor	Graphite	**0.014**	4.723	0.119
		GVAE	**0.014**	4.723	0.119
		GraphRNN	0.002	4.587	0.019
		WGAN	0.000	8.155	0.035
		CO-VAE	0.005	**4.220**	**0.006**
		DECO-VAE	0.002	4.588	0.019
	Transitivity	Graphite	0.010	4.663	0.088
		GVAE	0.010	4.663	0.088
		GraphRNN	0.020	5.671	0.074
		WGAN	0.000	8.009	0.047
		CO-VAE	0.010	**4.177**	0.006
		DECO-VAE	**0.025**	4.471	**0.004**

Based on the results in the main manuscripts, we exclude GAN from this supplementary analysis. The top panel of Fig. 2 shows <NAT-C>, the average NAT-C, and <NONNAT-C>, the average NONNAT-C, over generated contact graphs for each of the five models. The bottom panel focuses on CO-VAE and DECO-VAE. Table 3 lists the actual values for each metric for each model.

The distribution of NAT-C and NONNAT-C values over the training dataset are compared visually to the corresponding distributions over the data generated by DECO-VAE on three selected target proteins in Fig. 3.

Table 2. (Continued) Comparison of learned distributions (on each of the four graph properties of interest, listed in the rows) by their distance (according to PCC, BD, and EMD) to the training dataset for different proteins. The lowest BD and EMD values on each of the four graph properties of interest are highlighted in bold font.

	Graph Property	Methods	PCC	BD	EMD
D 5	Density	Graphite	0.005	4.475	0.404
		GVAE	0.018	4.408	0.404
		GraphRNN	0.008	4.952	0.163
		WGAN	0.000	6.788	0.021
		CO-VAE	0.008	3.837	**0.004**
		DECO-VAE	**0.028**	**3.800**	0.006
	N Edges	Graphite	0.005	4.4758	1033.676
		GVAE	0.018	4.408	1033.120
		GraphRNN	0.008	4.952	416.697
		WGAN	0.000	14.397	42.555
		CO-VAE	0.008	3.837	**11.160**
		DECO-VAE	**0.028**	**3.800**	16.085
	Avg-Deg-Cor	Graphite	0.005	4.837	0.142
		GVAE	**0.011**	4.849	0.141
		GraphRNN	0.009	5.162	0.322
		WGAN	0.000	8.260	**0.060**
		CO-VAE	0.003	**4.338**	0.064
		DECO-VAE	0.008	4.668	0.079
	Transitivity	Graphite	0.026	4.800	0.092
		GVAE	0.027	4.724	0.092
		GraphRNN	**0.028**	5.158	0.204
		WGAN	0.000	8.143	0.047
		CO-VAE	0.020	**4.454**	**0.004**
		DECO-VAE	0.004	4.559	0.005

	Graph Property	Methods	PCC	BD	EMD
D 6	Density	Graphite	**0.048**	2.532	0.393
		GVAE	0.027	2.269	0.393
		GraphRNN	0.027	3.848	0.006
		WGAN	0.000	5.051	0.005
		CO-VAE	0.000	**1.895**	0.002
		DECO-VAE	0.020	2.313	**0.000**
	N Edges	Graphite	**0.048**	2.532	842.692
		GVAE	0.027	2.268	843.290
		GraphRNN	0.027	3.818	13.864
		WGAN	0.000	12.722	10.403
		CO-VAE	0.000	**1.895**	3.769
		DECO-VAE	0.020	2.314	**1.492**
	Avg-Deg-Cor	Graphite	0.015	2.977	0.125
		GVAE	0.009	2.946	0.125
		GraphRNN	0.010	4.164	0.190
		WGAN	0.000	6.575	0.045
		CO-VAE	**0.057**	**2.713**	0.010
		DECO-VAE	0.022	2.918	**0.009**
	Transitivity	Graphite	0.061	2.988	0.089
		GVAE	0.019	2.981	0.089
		GraphRNN	**0.144**	4.292	0.260
		WGAN	0.000	6.418	0.050
		CO-VAE	0.026	**2.661**	**0.003**
		DECO-VAE	0.008	2.789	**0.003**

	Graph Property	Methods	PCC	BD	EMD
D 7	Density	Graphite	0.008	3.904	0.450
		GVAE	**0.027**	3.800	0.450
		GraphRNN	0.017	4.326	0.012
		WGAN	0.000	5.892	0.043
		CO-VAE	0.020	**2.895**	**0.009**
		DECO-VAE	0.006	3.219	0.012
	N Edges	Graphite	0.008	3.904	4314.204
		GVAE	**0.027**	3.800	4320.031
		GraphRNN	0.017	4.326	91.851
		WGAN	0.000	13.652	101.028
		CO-VAE	0.020	**2.878**	**89.220**
		DECO-VAE	0.006	3.219	111.235
	Avg-Deg-Cor	Graphite	0.000	4.082	0.218
		GVAE	0.008	4.083	0.218
		GraphRNN	0.000	4.240	0.247
		WGAN	0.000	7.445	**0.039**
		CO-VAE	0.000	**3.662**	0.189
		DECO-VAE	**0.014**	3.913	0.185
	Transitivity	Graphite	0.003	4.061	0.118
		GVAE	0.011	3.946	0.118
		GraphRNN	0.000	4.351	0.252
		WGAN	0.000	7.338	0.041
		CO-VAE	0.010	**3.615**	0.036
		DECO-VAE	**0.015**	3.810	**0.034**

	Graph Property	Methods	PCC	BD	EMD
D 8	Density	Graphite	0.009	3.598	0.392
		GVAE	0.009	3.598	0.393
		GraphRNN	0.011	4.227	0.007
		WGAN	0.000	6.081	0.005
		CO-VAE	0.024	**2.409**	**0.001**
		DECO-VAE	**0.026**	2.572	**0.001**
	N Edges	Graphite	0.009	3.598	1032.517
		GVAE	0.009	3.588	1032.520
		GraphRNN	0.011	4.227	17.749
		WGAN	0.000	13.871	11.234
		CO-VAE	0.024	**2.409**	**1.356**
		DECO-VAE	**0.026**	2.572	1.507
	Avg-Deg-Cor	Graphite	0.036	3.927	0.196
		GVAE	0.036	3.928	0.196
		GraphRNN	0.003	4.293	0.282
		WGAN	0.000	7.645	0.022
		CO-VAE	**0.044**	**3.591**	**0.010**
		DECO-VAE	0.018	3.990	**0.010**
	Transitivity	Graphite	0.004	4.010	0.055
		GVAE	0.004	4.009	0.055
		GraphRNN	0.007	4.844	0.231
		WGAN	0.000	7.532	0.041
		CO-VAE	**0.033**	**3.671**	**0.003**
		DECO-VAE	0.006	3.945	**0.003**

Table 4 lists the precision, coverage, recall, and F1 scores obtained by each model over the generated datasets, considering an experimentally-available structure as the ground truth. Fig. 4 highlights the F1 values obtained, excluding WGAN from this analysis (see manuscript).

Supplementary Table 5 compares the contact graphs generated by CO-VAE and DECO-VAE to the reference/training dataset and reports the differences in the average of NAT-C and NNONAT-C values obtained over the generated versus the training dataset.

We show the contact graphs and corresponding tertiary structures when varying latent factors for four more protein targets in Fig. 6 Fig. 7, respectively. The target proteins chosen are those with native PDB ids 1AOY, 1HHP, 1ISU(A), and 2H5N(D). The

Table 2. (Continued) Comparison of learned distributions (on each of the four graph properties of interest, listed in the rows) by their distance (according to PCC, BD, and EMD) to the training dataset for different proteins. The lowest BD and EMD values on each of the four graph properties of interest are highlighted in bold font.

	Graph Property	Methods	PCC	BD	EMD
D 9	Density	Graphite	0.008	5.410	0.465
		GVAE	0.007	5.415	0.466
		GraphRNN	**0.022**	**2.450**	0.004
		WGAN	0.000	5.594	0.061
		CO-VAE	0.018	4.941	**0.002**
		DECO-VAE	0.004	3.740	0.004
	Number of Edges	Graphite	0.007	5.410	1327.220
		GVAE	0.007	5.415	1328.796
		GraphRNN	**0.022**	**2.450**	9.030
		WGAN	0.000	13.495	164.424
		CO-VAE	0.019	4.941	**5.423**
		DECO-VAE	0.004	3.741	11.293
	Avg-Deg-Cor	Graphite	**0.030**	5.056	0.136
		GVAE	0.009	5.401	0.030
		GraphRNN	NA	3.413	NA
		WGAN	0.000	7.191	0.265
		CO-VAE	0.005	5.310	**0.017**
		DECO-VAE	0.004	**3.361**	0.092
	Transitivity	Graphite	0.008	5.436	0.244
		GVAE	0.008	5.348	0.307
		GraphRNN	**0.009**	**2.886**	0.122
		WGAN	0.000	7.133	0.205
		CO-VAE	0.005	5.159	**0.004**
		DECO-VAE	0.005	3.571	0.027
D 10	Density	Graphite	**0.026**	**3.526**	0.393
		GVAE	0.024	4.572	0.411
		GraphRNN	0.023	4.976	0.025
		WGAN	0.000	6.437	0.010
		CO-VAE	0.010	3.845	0.009
		DECO-VAE	0.011	3.962	**0.008**
	N Edges	Graphite	**0.026**	**3.526**	1031.645
		GVAE	0.024	4.572	1234.551
		GraphRNN	0.023	4.976	74.632
		WGAN	0.000	14.444	30.164
		CO-VAE	0.010	3.845	27.51
		DECO-VAE	0.011	3.962	**23.31**
	Avg-Deg-Cor	Graphite	0.009	4.869	0.197
		GVAE	0.011	4.659	0.156
		GraphRNN	0.009	4.993	0.179
		WGAN	0.000	8.093	**0.041**
		CO-VAE	**0.012**	**4.286**	0.089
		DECO-VAE	0.005	4.455	0.061
	Transitivity	Graphite	0.004	**4.047**	0.055
		GVAE	**0.018**	4.718	0.093
		GraphRNN	0.009	5.071	0.258
		WGAN	0.000	7.971	0.063
		CO-VAE	0.001	4.231	**0.013**
		DECO-VAE	0.006	4.443	0.016
D 11	Density	Graphite	0.005	4.760	0.412
		GVAE	0.017	3.983	0.412
		GraphRNN	0.005	4.746	0.009
		WGAN	0.000	5.843	**0.008**
		CO-VAE	**0.020**	3.198	0.023
		DECO-VAE	0.006	**3.005**	0.026
	N Edges	Graphite	0.005	4.762	1799.794
		GVAE	0.017	3.983	1400.567
		GraphRNN	0.005	4.746	**33.934**
		WGAN	0.000	13.976	26.482
		CO-VAE	**0.020**	3.198	79.542
		DECO-VAE	0.006	**3.005**	88.277
	Avg-Deg-Cor	Graphite	0.001	5.045	0.199
		GVAE	**0.035**	3.939	0.110
		GraphRNN	0.020	4.617	0.213
		WGAN	0.000	7.401	**0.050**
		CO-VAE	0.000	3.555	0.112
		DECO-VAE	0.004	**3.543**	0.106
	Transitivity	Graphite	0.002	4.944	0.115
		GVAE	0.023	3.999	**0.079**
		GraphRNN	0.005	4.640	0.311
		WGAN	0.000	7.288	0.080
		CO-VAE	0.016	3.347	0.089
		DECO-VAE	**0.025**	**3.283**	0.129
D 12	Density	Graphite	0.004	4.753	0.411
		GVAE	0.004	4.754	0.411
		GraphRNN	**0.015**	5.069	0.003
		WGAN	0.000	7.703	0.655
		CO-VAE	0.005	**4.076**	**0.002**
		DECO-VAE	0.010	4.187	0.001
	N Edges	Graphite	0.004	4.754	1796.572
		GVAE	0.004	4.754	1796.570
		GraphRNN	**0.015**	5.069	13.888
		WGAN	0.000	15.953	2507.665
		CO-VAE	0.005	**4.076**	**8.554**
		DECO-VAE	0.010	4.184	5.195
	Avg-Deg-Cor	Graphite	0.000	5.045	0.198
		GVAE	0.001	5.045	0.199
		GraphRNN	**0.013**	5.221	0.216
		WGAN	0.000	8.558	0.197
		CO-VAE	0.004	**4.516**	0.024
		DECO-VAE	0.001	4.884	**0.016**
	Transitivity	Graphite	**0.019**	4.899	0.115
		GVAE	0.009	4.898	0.115
		GraphRNN	0.011	5.247	0.316
		WGAN	0.000	8.498	0.260
		CO-VAE	0.004	**4.575**	**0.002**
		DECO-VAE	0.004	4.859	**0.002**

latent factor we isolate to vary demonstrates its impact/control on the contact graphs (and corresponding tertiary structures). For example, on 1AOY, one can see that the factor controls the placement of the other secondary structures with respect to the major one in orange. On 1HHP, a core has been formed, and the factor largely controls the placement of the helix in green in relation to the core. On 1ISU(A), the factor largely controls the placement of the rest of the elements around the helix in green. One 2H5N(D), the factor largely controls the unfolding/folding of a core region and the packing of the helix in magenta around the core.

Table 2. (Continued) Comparison of learned distributions (on each of the four graph properties of interest, listed in the rows) by their distance (according to PCC, BD, and EMD) to the training dataset for different proteins. The lowest BD and EMD values on each of the four graph properties of interest are highlighted in bold font.

	Graph Property	Methods	PCC	BD	EMD
D 13	Density	Graphite	0.012	2.744	0.421
		GVAE	0.033	2.857	0.419
		GraphRNN	**0.069**	3.893	0.012
		WGAN	0.000	4.788	0.008
		CO-VAE	0.022	**1.939**	**0.003**
		DECO-VAE	0.000	2.569	**0.003**
	N Edges	Graphite	0.012	2.744	2044.417
		GVAE	0.033	2.857	2033.940
		GraphRNN	**0.069**	3.893	57.191
		WGAN	0.000	13.275	37.082
		CO-VAE	0.022	**1.939**	**12.213**
		DECO-VAE	0.000	2.569	15.585
	Avg-Deg-Cor	Graphite	**0.058**	3.094	0.191
		GVAE	0.044	3.098	0.190
		GraphRNN	0.001	4.095	0.260
		WGAN	0.000	6.584	0.056
		CO-VAE	0.006	**2.731**	0.032
		DECO-VAE	0.027	2.872	**0.024**
	Transitivity	Graphite	**0.031**	3.120	0.119
		GVAE	0.014	3.126	0.119
		GraphRNN	0.005	4.269	0.282
		WGAN	0.000	6.465	0.067
		CO-VAE	0.019	**2.771**	0.004
		DECO-VAE	0.002	2.898	**0.003**

	Graph Property	Methods	PCC	BD	EMD
D 14	Density	Graphite	0.002	1.711	0.440
		GVAE	0.059	1.896	0.426
		GraphRNN	0.004	3.544	**0.003**
		WGAN	0.000	3.501	0.005
		CO-VAE	**0.071**	**0.926**	0.006
		DECO-VAE	0.006	1.299	0.008
	N Edges	Graphite	0.002	1.711	3777.273
		GVAE	0.059	1.896	3735.452
		GraphRNN	0.004	3.544	**22.449**
		WGAN	0.000	12.424	40.487
		CO-VAE	**0.072**	**0.926**	55.227
		DECO-VAE	0.006	1.299	71.684
	Avg-Deg-Cor	Graphite	0.022	2.014	0.201
		GVAE	**0.057**	1.988	0.200
		GraphRNN	0.033	3.749	0.275
		WGAN	0.000	5.472	**0.016**
		CO-VAE	0.004	**1.439**	0.060
		DECO-VAE	0.019	1.811	0.060
	Transitivity	Graphite	0.032	1.993	0.107
		GVAE	0.072	2.000	0.107
		GraphRNN	**0.085**	3.720	0.313
		WGAN	0.000	5.348	0.036
		CO-VAE	0.038	**1.316**	**0.022**
		DECO-VAE	0.002	1.540	0.024

	Graph Property	Methods	PCC	BD	EMD
D 15	Density	Graphite	**0.034**	3.249	0.445
		GVAE	0.006	3.272	0.444
		GraphRNN	0.001	4.395	**0.002**
		WGAN	0.000	4.628	0.004
		CO-VAE	0.001	3.022	0.022
		DECO-VAE	0.009	**2.293**	0.021
	N Edges	Graphite	**0.034**	3.249	4705.846
		GVAE	0.006	3.274	4694.770
		GraphRNN	0.001	4.395	**16.850**
		WGAN	0.000	13.895	40.345
		CO-VAE	0.001	3.022	229.12
		DECO-VAE	0.009	**2.293**	217.25
	Avg-Deg-Cor	Graphite	0.012	3.292	0.217
		GVAE	0.046	3.249	0.219
		GraphRNN	**0.053**	4.263	0.302
		GAN	0.000	6.596	**0.138**
		CO-VAE	0.024	3.165	0.180
		DECO-VAE	0.015	**3.067**	0.183
	Transitivity	Graphite	0.024	3.243	0.122
		GVAE	0.025	3.189	0.122
		GraphRNN	0.034	4.368	0.329
		WGAN	0.000	6.440	0.154
		CO-VAE	0.017	**2.819**	**0.113**
		DECO-VAE	**0.043**	2.944	0.118

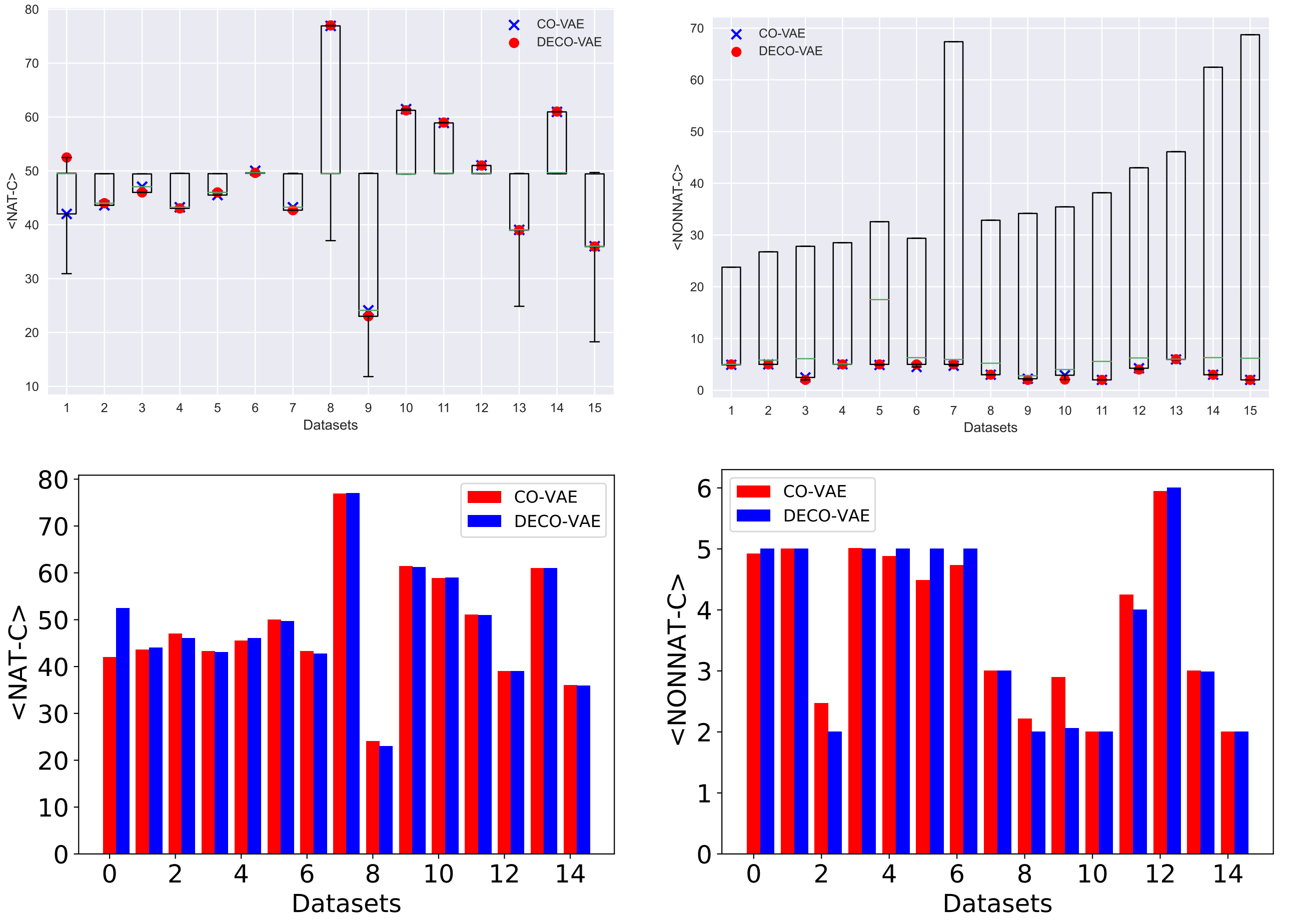

Fig. 2. The generated graphs are compared to a reference graph computed over an experimentally-available structure for each protein, and <NAT-C> and <NONNAT-C> values are computed over generated graphs. The boxplots show the range over all five models across all datasets. Annotations denote the values obtained by CO-VAE and DECO-VAE.

Table 3. Evaluation of the average of the NAT-C and NONNAT-C metrics over generated contact graphs. The best value in each metric (on each protein target) is highlighted in bold font. G-RNN abbreviates GraphRNN. Column 2 shows the PDB id (five-letter code to locate entries in the PDB at rcsb.org) of an experimentally-available structure employed as reference.

Dataset	PDB ID	Metric	VGAE	Graphite	G-RNN	WGAN	CO-VAE	DECO-VAE
1.	1BQ9	<NAT-C>	49.49	**49.56**	30.92	1.96	42.01	42.48
		<NONNAT-C>	23.79	23.79	**4.88**	7.98	4.92	5.00
2.	1DTD(B)	<NAT-C>	49.47	**49.48**	26.82	1.96	43.61	44.00
		<NONNAT-C>	26.75	26.75	5.82	8.88	**5.00**	**5.00**
3.	1ISU(A)	<NAT-C>	49.43	**49.45**	28.65	12.11	47.06	46.00
		<NONNAT-C>	27.82	27.84	6.10	56.04	2.47	**2.00**
4.	1C8C(A)	<NAT-C>	49.52	**49.54**	29.45	2.09	43.24	43.04
		<NONNAT-C>	28.53	28.52	5.06	8.58	5.01	**5.00**
5.	1HZ6(A)	<NAT-C>	49.48	**49.50**	35.48	1.67	45.51	46.00
		<NONNAT-C>	32.59	32.58	17.52	7.75	**4.88**	5.00
6.	1SAP	<NAT-C>	49.56	49.67	30.35	2.04	**50.02**	49.63
		<NONNAT-C>	29.37	29.38	6.30	8.63	**4.49**	5.00
7.	1FWP	<NAT-C>	49.46	**49.55**	24.26	1.27	43.24	42.71
		<NONNAT-C>	67.38	67.37	5.94	6.07	**4.73**	5.00
8.	1AIL	<NAT-C>	49.51	49.50	37.05	2.05	76.91	**77.00**
		<NONNAT-C>	32.89	32.86	5.22	8.94	**3.00**	**3.00**
9.	1DTJ(A)	<NAT-C>	**49.58**	49.52	11.81	0.98	24.11	23.01
		<NONNAT-C>	34.17	34.19	2.80	4.49	2.22	**2.00**
10.	1AOY	<NAT-C>	49.43	49.41	28.08	2.07	**61.46**	61.24
		<NONNAT-C>	35.44	35.44	4.00	8.35	2.90	**2.06**
11.	1CC5	<NAT-C>	49.57	49.51	30.32	1.71	58.90	**58.97**
		<NONNAT-C>	38.19	38.17	5.57	8.72	**2.00**	**2.00**
12.	1TIG	NAT-C/L	49.49	49.56	25.57	10.21	**51.02**	51.00
		<NONNAT-C>	43.00	43.00	6.24	46.31	4.25	**4.00**
13.	1HHP	NAT-C/L	49.47	**49.53**	24.86	2.12	39.05	39.00
		<NONNAT-C>	46.10	46.14	**5.91**	9.22	5.95	6.00
14.	2H5ND	NAT-C/L	49.46	49.67	26.33	2.00	60.95	**61.01**
		<NONNAT-C>	62.44	62.45	6.31	9.00	3.00	**2.99**
15.	1ALY	<NAT-C>	49.44	**49.70**	18.27	1.63	36.00	35.95
		<NONNAT-C>	68.72	68.72	6.18	7.99	**2.00**	**2.00**

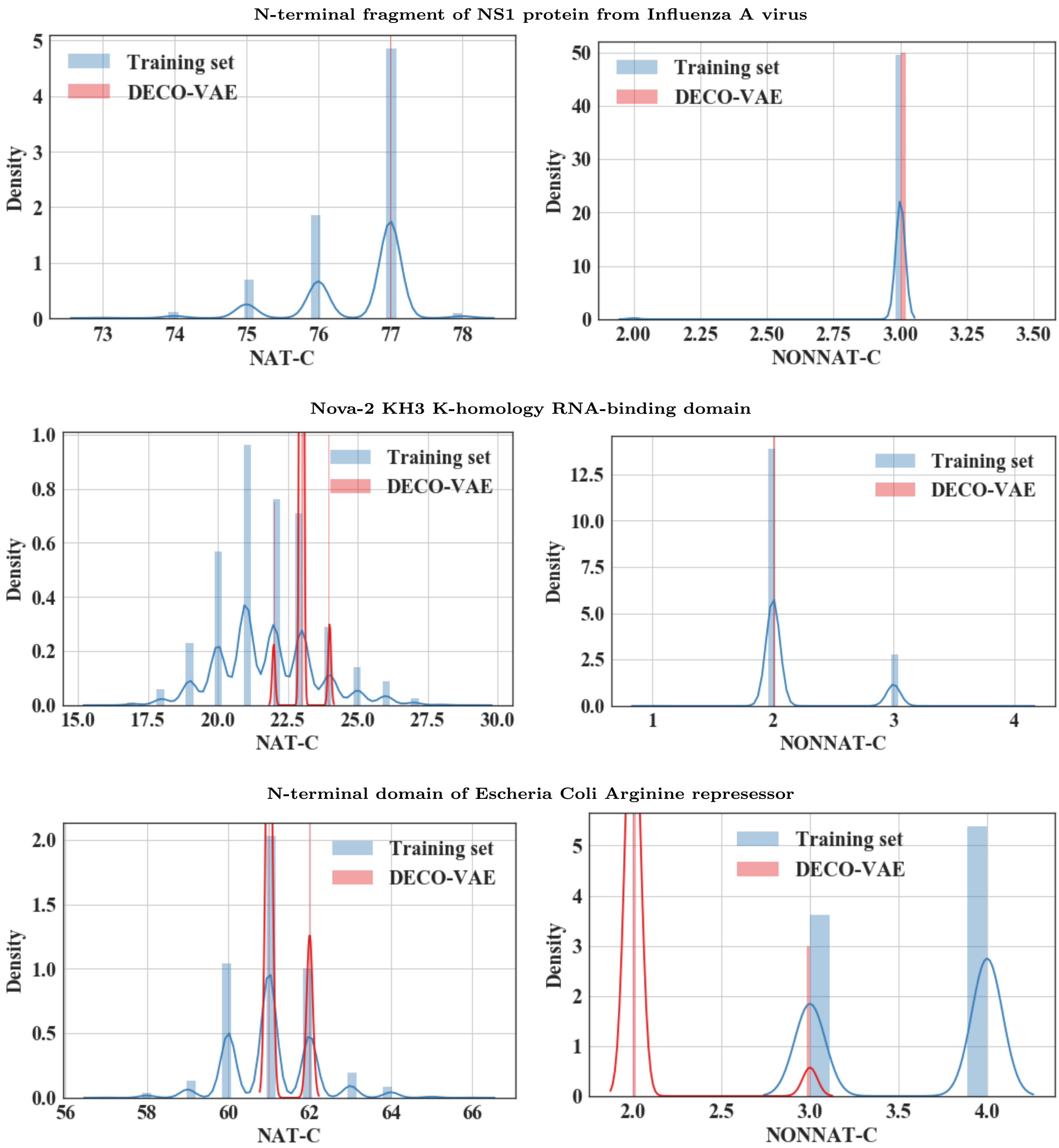

Fig. 3. Distributions of percentage of native contacts and number of non-native contacts per amino acid are shown for the DECO-VAE- and Rosetta-generated datasets for three selected protein targets. Each column contains both NAT-C and NONNAT-C distributions.

Table 4. Evaluation of generated contact graphs on each protein target across the five models in terms of precision, recall, coverage, and F1 score. The best value in each metric (on each protein target) is highlighted in bold font. Column 1 also shows the PDB id (five-letter code to locate entries in the PDB at rcsb.org of an experimentally-available structure employed as the ground truth.

PDB ID	Model	Prec	Covg	Rec	F1
1. 1BQ9	Graphite	0.1566	0.4809	0.4994	0.2384
	GVAE	0.1568	**0.4815**	0.5001	0.2387
	GraphRNN	0.3659	0.3232	0.3356	0.3494
	WGAN	0.2971	0.0447	**0.5817**	0.3932
	CO-VAE	**0.4658**	0.4451	0.4622	**0.4639**
	DECO-VAE	0.4638	0.4363	0.4531	0.4584
2. 1DTD(B)	Graphite	0.1526	0.4893	0.5002	0.2338
	GVAE	0.1526	0.4892	0.5001	0.2339
	GraphRNN	0.2974	0.2696	0.2755	0.2858
	WGAN	0.4336	0.0631	**0.8510**	0.5745
	CO-VAE	**0.6144**	0.5961	0.6093	0.6118
	DECO-VAE	0.6136	**0.6041**	0.6148	**0.6142**
3. 1ISU(A)	Graphite	0.1534	0.4891	0.4996	0.2348
	GVAE	0.1537	**0.4898**	0.5003	0.2351
	GraphRNN	0.2837	0.2546	0.2601	0.2711
	WGAN	0.0785	0.0709	**0.9501**	0.1450
	CO-VAE	0.7542	0.4738	0.4839	0.5886
	DECO-VAE	**0.8435**	0.4738	0.4839	**0.6151**
4. 1C8C(A)	Graphite	0.1322	0.4907	0.5002	0.2091
	GVAE	0.1319	0.4900	0.4995	0.2088
	GraphRNN	0.3381	0.3065	0.3124	0.3239
	WGAN	0.4300	0.0576	**0.8975**	0.5813
	CO-VAE	0.6513	**0.6707**	0.6837	0.6670
	DECO-VAE	**0.6538**	0.6695	0.6824	**0.6678**
5. 1HZ6(A)	Graphite	0.1500	**0.4933**	**0.5000**	0.2308
	GVAE	0.1500	**0.4933**	**0.5000**	0.2307
	GraphRNN	0.1849	0.3543	0.3592	0.2441
	WGAN	0.2781	0.0336	0.4641	0.3477
	CO-VAE	0.5960	0.4519	0.4581	0.5179
	DECO-VAE	**0.6067**	0.4545	0.4607	**0.5237**
6. 1SAP	Graphite	0.1329	0.4958	0.4993	0.2099
	GVAE	0.1332	0.4964	0.5000	0.2103
	GraphRNN	0.3021	0.3178	0.3202	0.3107
	WGAN	0.4341	0.0568	**0.8860**	0.5826
	CO-VAE	0.3021	0.3179	0.3201	0.3106
	DECO-VAE	**0.6681**	**0.6838**	0.6888	**0.6783**
7. 1FWP	Graphite	0.1405	0.4849	0.5001	0.2193
	GVAE	0.1401	0.4838	0.4989	0.2188
	GraphRNN	0.3478	0.2558	0.2638	0.2992
	WGAN	0.3644	0.0320	0.4610	0.4067
	CO-VAE	**0.8426**	**0.4705**	**0.4852**	**0.6158**
	DECO-VAE	0.8322	0.4695	0.4842	0.6122
8. 1AIL	Graphite	0.1277	0.4912	0.4994	0.2034
	GVAE	0.1276	0.4911	0.4992	0.2032
	GraphRNN	0.3900	0.3718	0.3780	0.3836
	WGAN	0.4545	0.0580	**0.9322**	0.6110
	CO-VAE	**0.8244**	0.8391	0.8531	**0.8385**
	DECO-VAE	0.8231	**0.8393**	0.8533	0.8379

PDB ID	Model	Prec	Covg	Rec	F1
9. 1DTJ(A)	Graphite	0.1320	0.5000	**0.5000**	0.2031
	GVAE	0.1390	**0.5109**	**0.5000**	0.2101
	GraphRNN	0.3153	0.1301	0.1301	0.1733
	WGAN	0.4449	0.0270	0.4373	0.4402
	CO-VAE	**0.7432**	0.3310	0.3312	0.4607
	DECO-VAE	0.7429	0.3347	0.3346	**0.4618**
10. 1AOY	Graphite	0.1065	0.4963	0.4995	0.1756
	GVAE	0.1066	0.4964	0.4996	0.1757
	GraphRNN	0.3717	0.2959	0.2978	0.3299
	WGAN	0.3908	0.0418	**0.8100**	0.5271
	CO-VAE	0.7620	**0.7363**	0.7409	0.7511
	DECO-VAE	**0.8109**	0.7299	0.7345	**0.7708**
11. 1CC5	Graphite	0.1234	0.4843	0.4997	0.1980
	GVAE	0.1234	0.4846	0.4999	0.1979
	GraphRNN	0.3489	0.2733	0.2820	0.3117
	WGAN	0.3771	0.0396	**0.6448**	0.4757
	CO-VAE	**0.8972**	**0.5878**	0.6064	**0.7237**
	DECO-VAE	0.8965	0.5864	0.6051	0.7225
12. 1TIG	Graphite	0.1055	0.4945	0.5008	0.1742
	GVAE	0.1054	0.4939	0.5001	0.1740
	GraphRNN	0.2787	0.2687	0.2721	0.2752
	WGAN	0.0544	0.0286	0.5555	0.0991
	CO-VAE	0.5797	**0.5691**	**0.5764**	**0.5780**
	DECO-VAE	**0.5799**	0.5689	0.5761	**0.5780**
13. 1HHP	Graphite	0.0902	0.4979	0.5003	0.1529
	GVAE	0.0903	0.4978	0.5002	0.1529
	GraphRNN	0.2804	0.2582	0.2594	0.2693
	WGAN	0.4040	0.0376	**0.8554**	0.5488
	CO-VAE	0.6494	**0.6612**	0.6643	0.6567
	DECO-VAE	**0.6544**	0.6591	0.6622	**0.6583**
14. 2H5N(D)	Graphite	0.0748	0.4976	0.5004	0.1302
	GVAE	0.0747	0.4971	0.4998	0.1300
	GraphRNN	0.2846	0.2829	0.2845	0.2844
	WGAN	0.3195	0.0233	0.6364	0.4250
	CO-VAE	0.7127	**0.6393**	**0.6428**	0.6759
	DECO-VAE	**0.7248**	0.6354	0.6389	**0.6791**
15. 1ALY	Graphite	0.0684	0.4981	0.5002	0.1203
	GVAE	0.0683	**0.4989**	**0.5011**	0.1206
	GraphRNN	0.2356	0.1979	0.1987	0.2155
	WGAN	0.2607	0.0142	0.4235	0.3226
	CO-VAE	0.8034	0.4463	0.4482	0.5754
	DECO-VAE	**0.8468**	0.4408	0.4427	**0.5791**

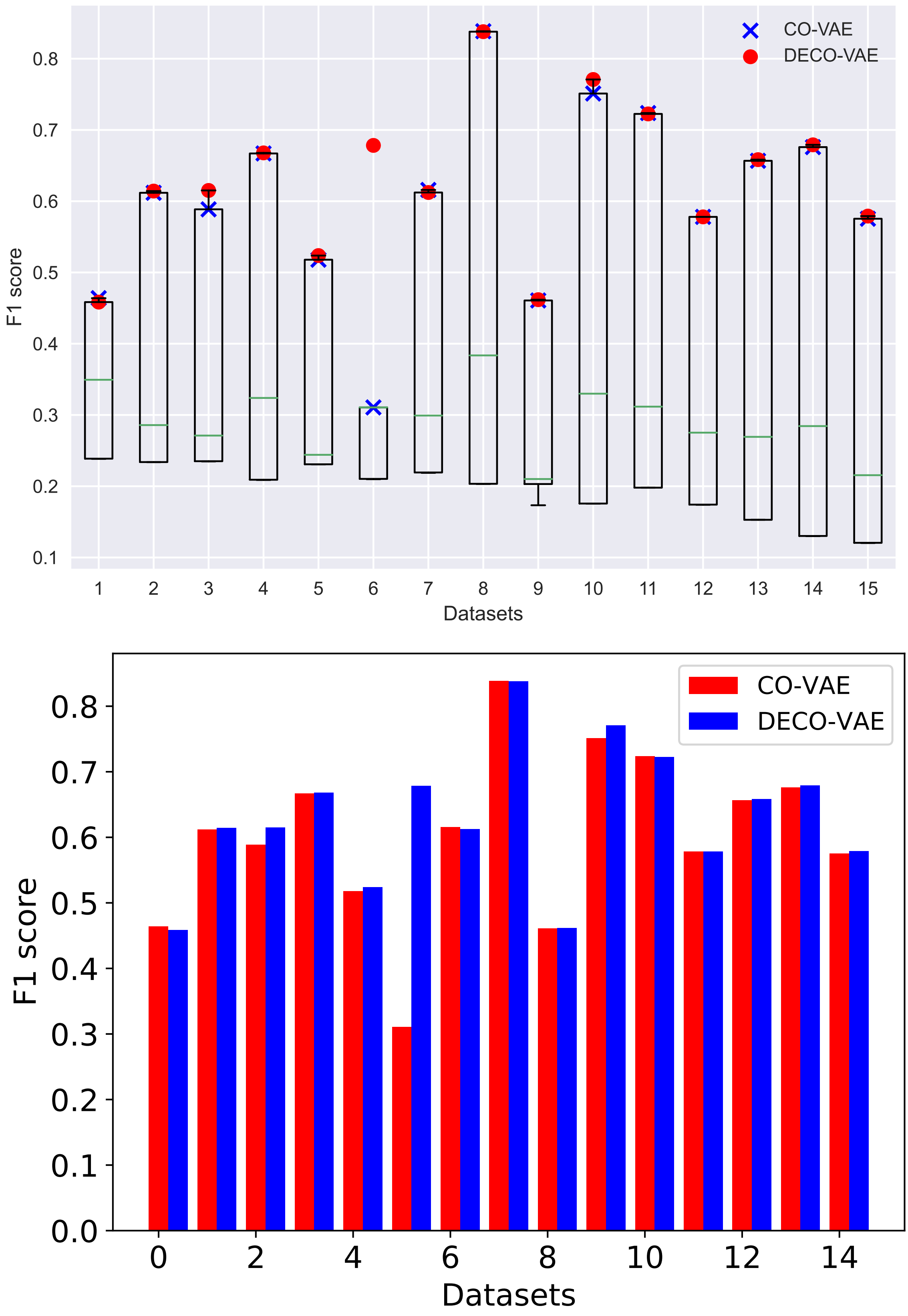

Fig. 4. The generated graphs are compared to a reference graph computed over an experimentally-available structure for each protein. The top panel shows via boxplots the range of F1 scores over all five models, excluding WGAN, across all datasets. Annotations denote the values obtained by CO-VAE and DECO-VAE. The bottom panel focuses the comparison to CO-VAE and DECO-VAE.

Table 5. Comparison of the average of the <NAT-C> and <NONNAT-C> metrics over contact graphs generated by CO-VAE and DECO-VAE to those over the training datasets. Column 2 reports on the difference (Δ) of each metric in the CO-VAE- or DECO-VAE-generated datasets from the corresponding metric in the training datasets. Smallest values per metric on each protein target are highlighted in bold font. Column 2 shows the PDB id (five-letter code to locate entries in the PDB at rcsb.org of an experimentally-available structure employed as reference.

Dataset	PDB ID	Metric	CO-VAE	DECO-VAE
Dataset 1	1BQ9	Δ-NAT	**0.48**	0.95
		Δ-NONNAT	**0.01**	0.08
Dataset 2	1DTD(B)	Δ-NAT	**0.19**	0.57
		Δ-NONNAT	**0.01**	**0.01**
Dataset 3	1ISU(A)	Δ-NAT	**0.33**	0.73
		Δ-NONNAT	**2.43**	2.80
Dataset 4	1C8C(A)	Δ-NAT	0.23	**0.03**
		Δ-NONNAT	**0.01**	0.02
Dataset 5	1HZ6(A)	Δ-NAT	0.24	**0.01**
		Δ-NONNAT	0.28	**0.16**
Dataset 6	1SAP	Δ-NAT	0.36	**0.03**
		Δ-NONNAT	**0.03**	0.48
Dataset 7	1FWP	Δ-NAT	0.55	**0.02**
		Δ-NONNAT	1.40	**1.13**
Dataset 8	1AIL	Δ-NAT	**0.01**	**0.01**
		Δ-NONNAT	**0.01**	**0.01**
Dataset 9	1DTJ(A)	Δ-NAT	2.34	**1.24**
		Δ-NONNAT	**0.05**	0.17
Dataset 10	1AOY	Δ-NAT	0.39	**0.17**
		Δ-NONNAT	**0.69**	1.54
Dataset 11	1CC5	Δ-NAT	**1.97**	2.04
		Δ-NONNAT	**2.61**	**2.61**
Dataset 12	1TIG	Δ-NAT	**0.14**	0.16
		Δ-NONNAT	**0.31**	0.56
Dataset 13	1HHP	Δ-NAT	0.22	**0.17**
		Δ-NONNAT	0.06	**0.01**
Dataset 14	2H5ND	Δ-NAT	0.07	**0.01**
		Δ-NONNAT	**0.93**	0.94
Dataset 15	1ALY	Δ-NAT	0.47	**0.42**
		Δ-NONNAT	**2.99**	**2.99**

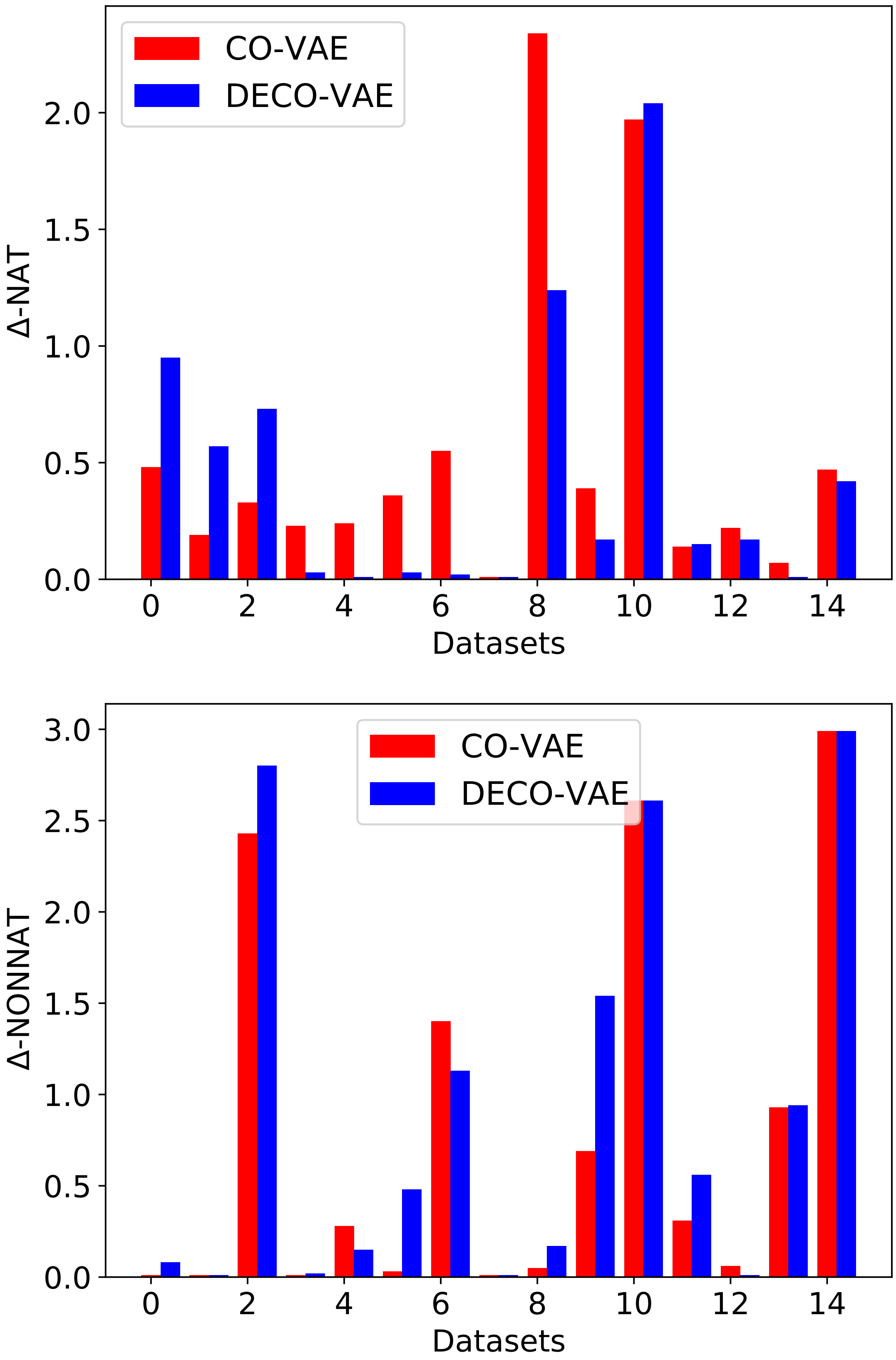

Fig. 5. Comparison of the <NAT-C> and <NONNAT-C> values, respectively, over contact graphs generated by CO-VAE and DECO-VAE to those in the training datset, reporting the difference (δ).

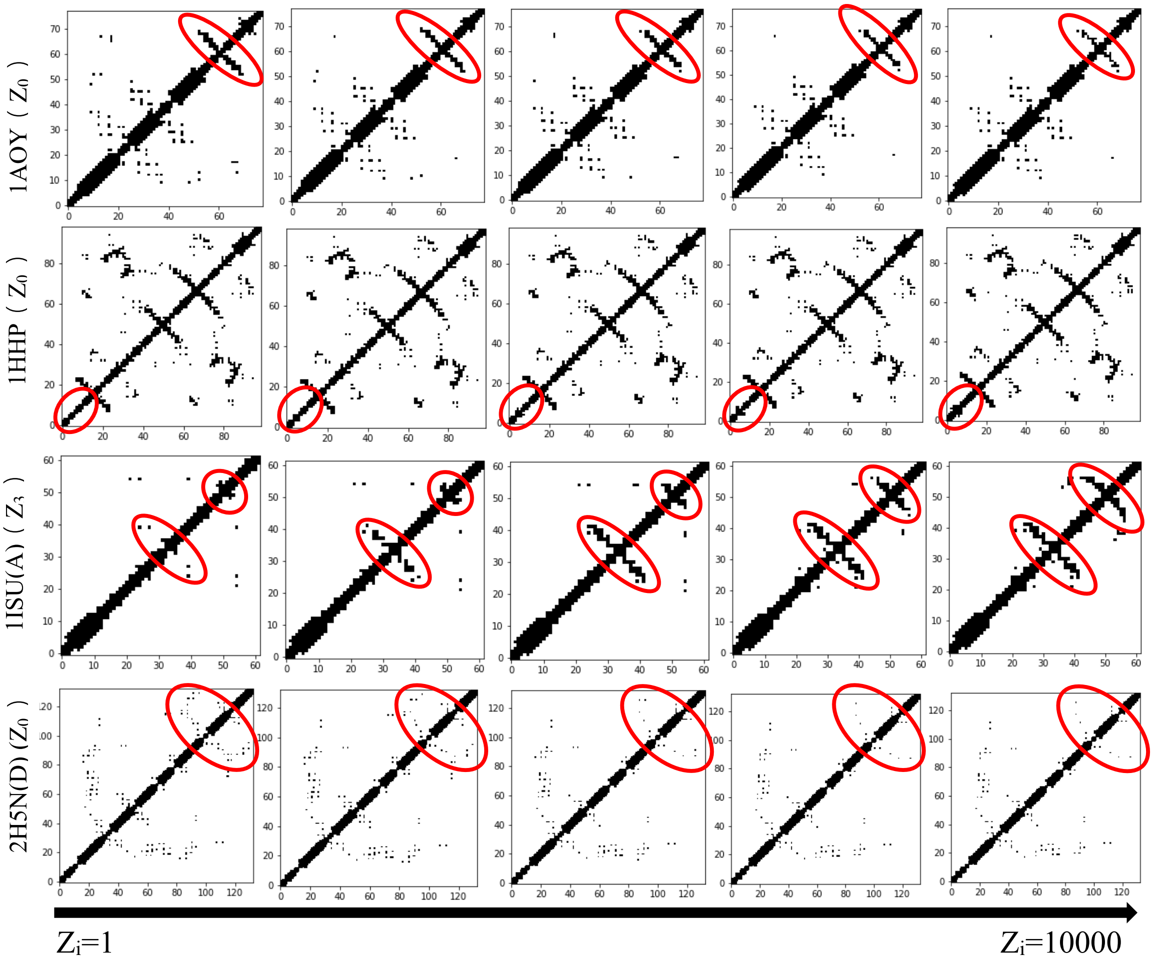

Fig. 6. Generated contact graphs for four more selected protein targets (with native PDB ids 1AOY, 1HHP, 1ISU(A), and 2H5N(D)). For each, one of the semantic factors discovered in the latent variables is varied as above to visualize changes in contacts.

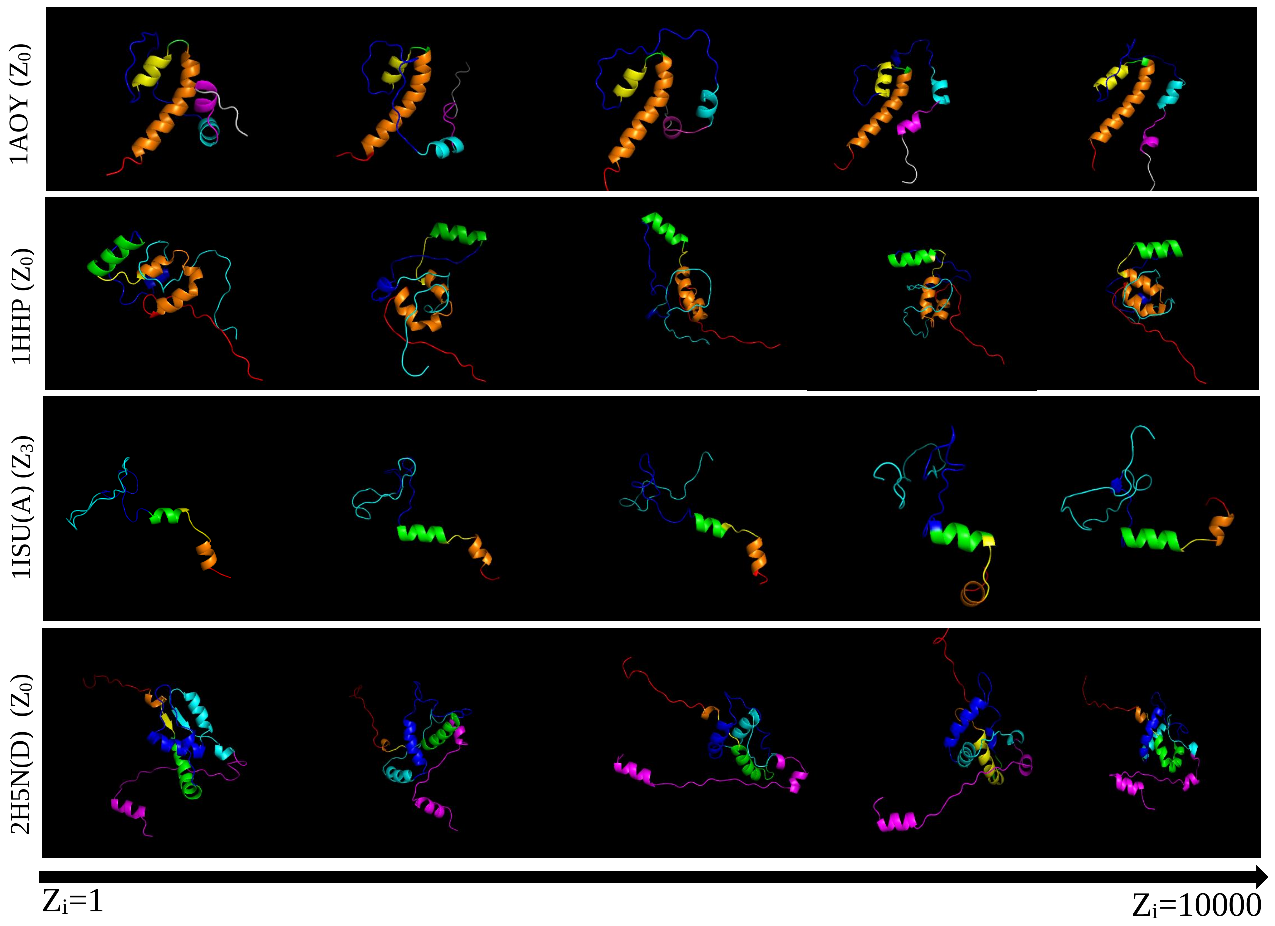

Fig. 7. Tertiary protein structures corresponding to the contact graphs related in Fig 6. Secondary structures are drawn in different colors.
